# Supplementary material for: Antimicrobial Lavandulylated Flavonoids from a Sponge-Derived Streptomyces sp. G248 in East Vietnam Sea
Source: Mar Drugs. 2019 Sep 10;17(9):529. doi: 10.3390/md17090529 (PMC6780759; doi:10.3390/md17090529)
Supplement: Supplementary file 1 [file marinedrugs-17-00529-s001.pdf]

SUPPLEMENTARY MATERIAL

Antimicrobial Lavandulylated Flavonoids from a Sponge-Derived *Streptomyces* sp. G248 in East Vietnam Sea

Duc Danh Cao[1,2], Thi Thanh Van Trinh[1], Huong Doan Thi Mai[1,2], Van Nam Vu[1], Thi Hong Minh Le[1], Quyen Vu Thi[1], Mai Anh Nguyen[1], Thu Trang Duong[1], Dang Thach Tran[3], Van Minh Chau[1], Rui Ma[4], Gauri Shetye[4], Sanghyun Cho[4], Brian T. Murphy[5] and Van Cuong Pham[1,*]

[1] Advanced Center for Bioorganic Chemistry, Institute of Marine Biochemistry, Vietnam Academy of Science and Technology (VAST), Hanoi, Vietnam.
[2] Faculty of Chemistry, Graduate University of Science and Technology, VAST, Hanoi, Vietnam.
[3] Institute of Applied Science and Technology, University Industry Vinh, Vinh, Vietnam.
[4] Institute for Tuberculosis Research, College of Pharmacy, University of Illinois at Chicago, Chicago, USA.
[5] Department of Pharmaceutical Sciences, College of Pharmacy, University of Illinois at Chicago, Chicago, USA.
* Correspondence: phamvc@imbc.vast.vn; Tel.: +84-2437-9170-49 (V.C.P.).

Content

TD-DFT electronic CD (ECD) calculation of compounds **1 - 3**	S-4
Streptomyces sp. G248 isolation and characterization	S-5
Table 1S. Conformational population (%) for the most stable conformers of (2*S*,2″*R*)-**1**	S-8
Figure 4S. Optimized conformers of (2*S*,2″*R*)-**1**	S-8
Table 2S. Conformational population (%) for the most stable conformers of (2*S*,2″*S*)-**1**	S-9
Figure 5S. Optimized conformers of (2*S*,2″*S*)-**1**	S-9
Table 3S. Conformational population (%) for the most stable conformers of (2*S*,2″*R*)-**2**	S-10
Figure 6S. Optimized conformers of (2*S*,2″*R*)-**2**	S-10
Table 4S. Conformational population (%) for the most stable conformers of (2*S*,2″*S*)-**2**	S-11
Figure 7S. Optimized conformers of (2*S*,2″*S*)-**2**	S-11
Table 5S. Conformational population (%) for the most stable conformers of (2″*S*)-**3**	S-12
Figure 8S. Optimized conformers of (2″*S*)-**3**	S-12
Figure 9S. ^{1}H NMR spectrum of **1** (500 MHz, CD_3OD)	S-13
Figure 10S. ^{13}C NMR spectrum of **1** (125 MHz, CD_3OD)	S-14
Figure 11S. ^{1}H-^{1}H COSY spectrum of **1** (500 MHz, CD_3OD)	S-15
Figure 12S. HSQC spectrum of **1** (^{1}H: 500 MHz, ^{13}C: 125 MHz, CD_3OD)	S-16
Figure 13S. HMBC spectrum of **1** (^{1}H: 500 MHz, ^{13}C: 125 MHz, CD_3OD)	S-17
Figure 14S. ROESY spectrum of **1** (500 MHz, CD_3OD)	S-18
Figure 15S. HR-ESI-MS of **1**	S-19
Figure 16S. IR spectrum of **1**	S-20
Figure 17S. CD spectrum of **1**	S-21
Figure 18S. ^{1}H NMR spectrum of **2** (500 MHz, CD_3OD)	S-22
Figure 19S. ^{13}C NMR spectrum of **2** (125 MHz, CD_3OD)	S-23
Figure 20S. ^{1}H-^{1}H COSY spectrum of **2** (500 MHz, CD_3OD)	S-24
Figure 21S. HSQC spectrum of **2** (^{1}H: 500 MHz, ^{13}C: 125 MHz, CD_3OD)	S-25
Figure 22S. HMBC spectrum of **2** (^{1}H: 500 MHz, ^{13}C: 125 MHz, CD_3OD)	S-26
Figure 23S. ^{1}H NMR spectrum of **2** (500 MHz, DMSO-d_6)	S-27
Figure 24S. ROESY spectrum of **2** (500 MHz, DMSO-d_6)	S-28
Figure 25S. HR-ESI-MS of **2**	S-29
Figure 26S. IR spectrum of **2**	S-30
Figure 27S. CD spectrum of **2**	S-31
Figure 28S. ^{1}H NMR spectrum of **3** (500 MHz, CD_3OD)	S-32
Figure 29S. ^{13}C NMR spectrum of **3** (125 MHz, CD_3OD)	S-33

Figure 30S. ^{1}H-^{1}H COSY spectrum of **3** (500 MHz, CD_3OD)	S-34
Figure 31S. HSQC spectrum of **3** (^{1}H: 500 MHz, ^{13}C: 125 MHz, CD_3OD)	S-35
Figure 32S. HMBC spectrum of **3** (^{1}H: 500 MHz, ^{13}C: 125 MHz, CD_3OD)	S-36
Figure 33S. ^{1}H-NMR spectrum of **3** (500 MHz**,** DMSO-d_6**)**	S-37
Figure 34S. ROESY spectrum of **3** (500 MHz, DMSO-d_6)	S-38
Figure 35S. HR-ESI-MS of **3**	S-39
Figure 36S. IR spectrum of **3**	S-40
Figure 37S. CD spectrum of **3**	S-41

TD-DFT electronic CD (ECD) calculation of compounds 1 - 3: A conformational search was carried out with Spartan'14 software (Wavefunction Inc., Irvine, CA) for the proposed absolute configuration as shown at the molecular mechanics level (MMFF) [1]. All MMFF minima were re-optimized using DFT calculations at the B3LYP/6-31G(d) level with the Gaussian 09 program [2]. The geometry was optimized starting from various initial conformations with vibrational frequency calculations confirming the presence of minima. The DFT calculations (B3LYP/LanL2DZ) were performed on the lowest-energy conformations using 30 excited states and a polarizable continuum model (PCM) in methanol. The ECD spectra of the conformers were combined using Boltzmann weighting with program SpecDis 1.71 (applying a sigma value of 0.25 eV) [3]. After applying a UV-shift correction, the computed CD spectra were compared with the CD curves experimentally obtained.

[1] Spartan'14. Wavefunction: Irvine, CA, USA, **2013**.

[2] Gaussian 09, Revision D.01, Frisch, M.J.; Trucks, G.W.; Schlegel, H.B.; Scuseria, G.E.; Robb, M.A.; Cheeseman, J.R.; Scalmani, G.; Barone, V.; Mennucci, B.; Petersson, G.A.; Nakatsuji, H.; Caricato, M.; Li, X.; Hratchian, H.P.; Izmaylov, A.F.; Bloino, J.; Zheng, G.; Sonnenberg, J.L.; Hada, M.; Ehara, M.; Toyota, K.; Fukuda, R.; Hasegawa, J.; Ishida, M.; Nakajima, T.; Honda, Y.; Kitao, O.; Nakai, H.; Vreven, T.; Montgomery, J.A., Jr.; Peralta, J.E.; Ogliaro, F.; Bearpark, M.; Heyd, J.J.; Brothers, E.; Kudin, K.N.; Staroverov, V.N.; Kobayashi, R.; Normand, J.; Raghavachari, K.; Rendell, A.; Burant, J.C.; Iyengar, S.S.; Tomasi, J.; Cossi, M.; Rega, N.; Millam, N.J.; Klene, M.; Knox, J.E.; Cross, J.B.; Bakken, V.; Adamo, C.; Jaramillo, J.; Gomperts, R.; Stratmann, R.E.; Yazyev, O.; Austin, A. J.; Cammi, R.; Pomelli, C.; Ochterski, J. W.; Martin, R.L.; Morokuma, K.; Zakrzewski, V.G.; Voth, G.A.; Salvador, P.; Dannenberg, J.J.; Dapprich, S.; Daniels, A.D.; Farkas, Ö.; Foresman, J.B.; Ortiz, J.V.; Cioslowski, J.; Fox, D.J. Gaussian, Inc., Wallingford CT, 2009.

[3] Bruhn, T.; Schaumlöffel, A.; Hemberger, Y.; Bringmann, G. SpecDis: Quantifying the comparison of calculated and experimental electronic circular dichroism spectra. *Chirality* **2013**, *25*, 243–249

***Streptomyces* sp. G248 isolation and characterization**

***Bacteria isolation*:** The sponge *Halichondria panicea* (Pallas, 1766) sample (0.5 g) was crushed by glass chopsticks in a falcon tube, to which sterile sea water (4.5 mL) was added. The mixture was homogenized by vortexing for 1 min, and the suspension was treated using a wet-heat technique (60 ºC for 6 min). An amount (0.5 mL) of this suspension was diluted by sterile distilled water (4.5 mL) and vortexed for 1 min, of which an aliquot of 50 µL was spread on A1 medium (soluble starch: 10 g/L, yeast extract: 4 g/L, peptone: 2 g/L, instant ocean: 30 g/L, agar: 15 g/L) supplemented with 50 µg/mL of polymycin B and cycloheximide to inhibit Gram (-) bacterial and fungal contaminations. After 21 days of aerobic incubation at 30 ºC, a colony of the actinomycete strain was transferred onto a petri dish of medium A1 for purification.

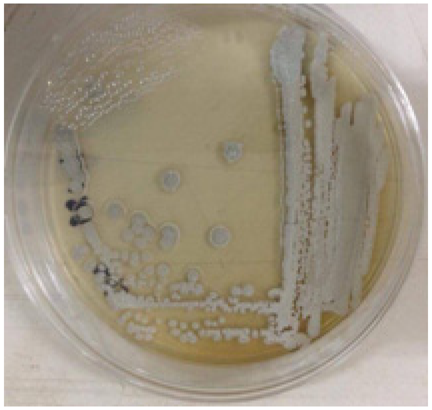

Figure 1S. Morphological appearance of strain G248

Bacteria identification: Genomic DNA was extracted with the Gen Elute Bacterial Genomic DNA kit (Sigma). The sequence of 16S rRNA was used for identification. The gene amplification was performed in a 25.0 µL mixture containing sdH_2O (16.3 µL), 10X PCR buffer (2.5 µL), 25 mM $MgCl_2$ (1.5 µL), 10 mM dNTP's (0.5 µL), Taq polymerase (0.2 µL), 0.05 mM (1.0 µL) for both primers of 9F (5'-GAGTTTGATCCTGGCTCAG3') and 1541R (5'-AAGGAGGTGATCCAACC3') and genomic DNA (2.0 µL). The reaction tube was then put into MJ Thermalcycler, which had been programmed to preheat at 94 ºC for 3 min, followed by 30 cycles of denaturation at 94 ºC for 1 min, annealing at 60 ºC for 30s and elongation at 72 ºC for 45s before a final extension of 72 ºC for 10 min. The estimated PCR

product size was about 1500 bp (Figure 2S). PCR products were purified by DNA purification kit (Invitrogen) then sequenced by DNA Analyzer (ABI PRISM 3100, Applied Bioscience). Gene sequences were handled by BioEdit v.2.7.5. and compared with bacterial 16S rRNA sequences in GeneBank database by NBCI Blast program. The results showed that the strain G248 belonged to the genus *Streptomyces*. The sequence of 16S rRNA gene of the strain G248 was registered on GenBank database with the accession number MG917690 (Figure 3S).

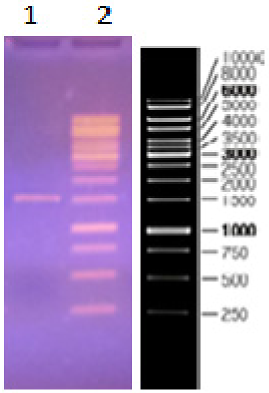

Figure 2S. Gel electrophoresis of PCR product

Lane 1: PCR product; Lane 2: One Kb DNA ladder of Fisher Scientific

Streptomyces sp. strain G248 16S ribosomal RNA gene, partial sequence

GenBank: MG917690.1

FASTA Graphics PopSet

Go to:

```
LOCUS       MG917690                1368 bp    DNA     linear   BCT 30-JUL-2018
DEFINITION  Streptomyces sp. strain G248 16S ribosomal RNA gene, partial
            sequence.
ACCESSION   MG917690
VERSION     MG917690.1
KEYWORDS    .
SOURCE      Streptomyces sp.
  ORGANISM  Streptomyces sp.
            Bacteria; Actinobacteria; Streptomycetales; Streptomycetaceae;
            Streptomyces.
REFERENCE   1  (bases 1 to 1368)
  AUTHORS   Le,T.H.M., Vu,T.Q., Nguyen,M.A., Pham,V.C., Doan,T.M.H., Tran,V.H.
            and Cao,D.T.
  TITLE     Partial 16S nRNA of strains isolated from the coastal in Viet Nam
  JOURNAL   Unpublished
REFERENCE   2  (bases 1 to 1368)
  AUTHORS   Le,T.H.M., Vu,T.Q., Nguyen,M.A., Pham,V.C., Doan,T.M.H., Tran,V.H.
            and Cao,D.T.
  TITLE     Direct Submission
  JOURNAL   Submitted (05-FEB-2018) Biotechnology, Institute of Marine
            Biochemistry, VAST, 18 Hoang Quoc Viet, Cau Giay, Ha Noi, Ha Noi
            10000, Viet Nam
COMMENT     ##Assembly-Data-START##
            Sequencing Technology :: Sanger dideoxy sequencing
            ##Assembly-Data-END##
FEATURES             Location/Qualifiers
     source          1..1368
```

Figure 3S. 16S rRNA gene sequence of *Streptomyces* sp. G248 on GenBank

Table 1S. Conformational population (%) for the most stable conformers of (2*S*,2″*R*)-**1**

Conformer	Population (%)
C-1	21.41
C-2	15.78
C-3	19.94
C-4	4.38
C-5	16.57
C-6	6.18
C-7	15.74

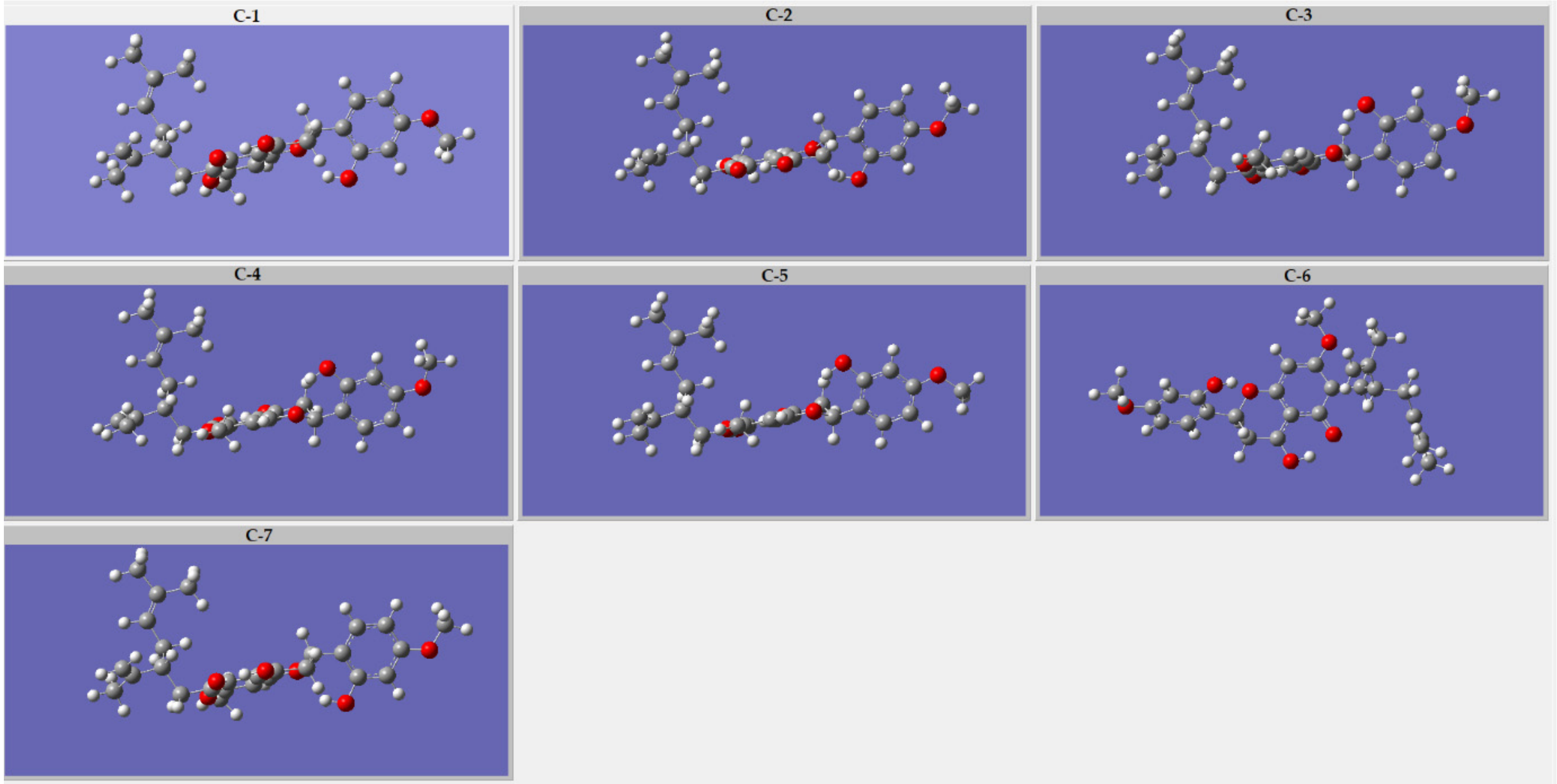

Figure 4S. Optimized conformers of (2*S*,2″*R*)-**1**

Table 2S. Conformational population (%) for the most stable conformers of (2*S*,2″*S*)-**1**

Conformer	Population (%)
C-1	13.76
C-2	8.83
C-3	19.86
C-4	12.64
C-5	3.05
C-6	10.71
C-7	6.92
C-8	15.22
C-9	9.02

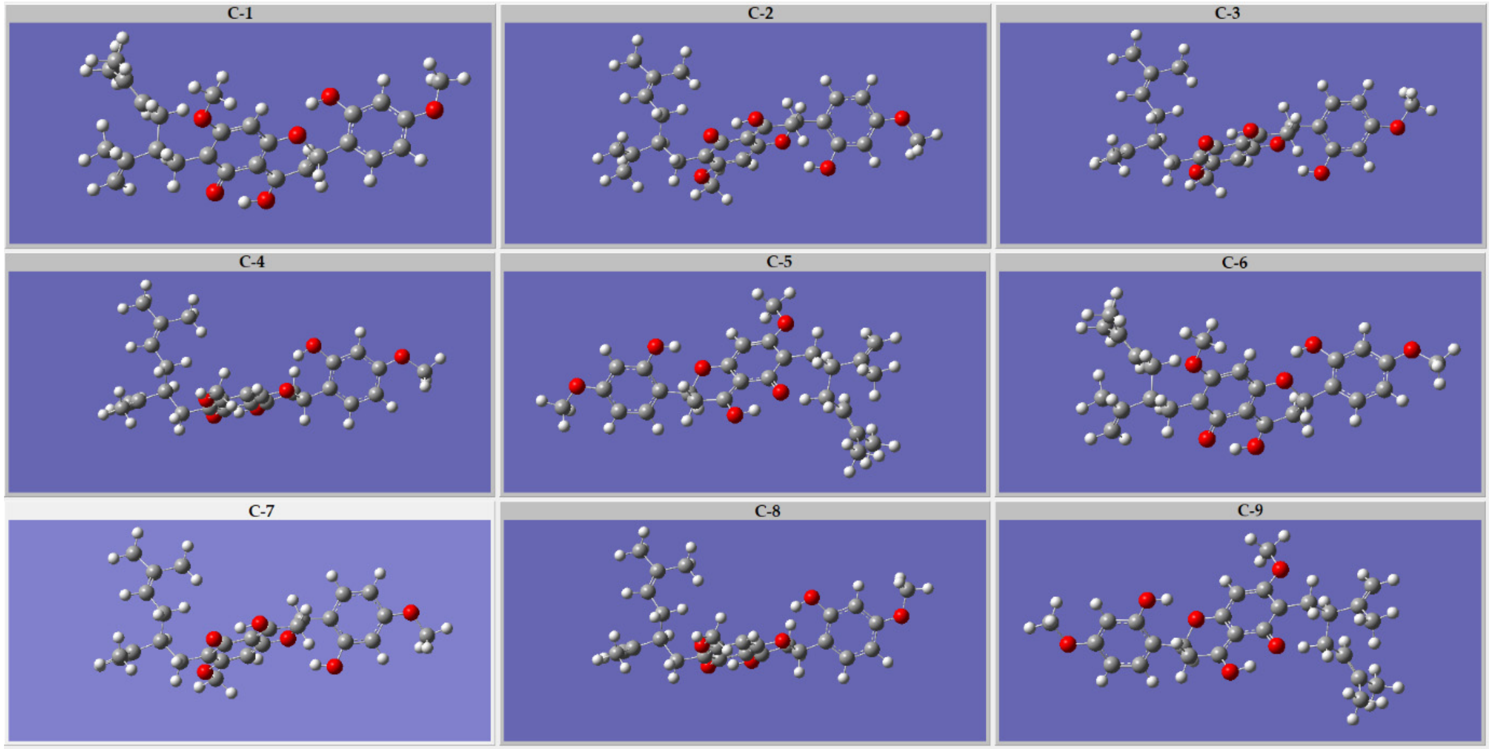

Figure 5S. Optimized conformers of (2*S*,2″*S*)-**1**

Table 3S. Conformational population (%) for the most stable conformers of (2*S*,2″*R*)-**2**

Conformer	Population (%)
C-1	8.54
C-2	36.15
C-3	8.85
C-4	8.72
C-5	37.73

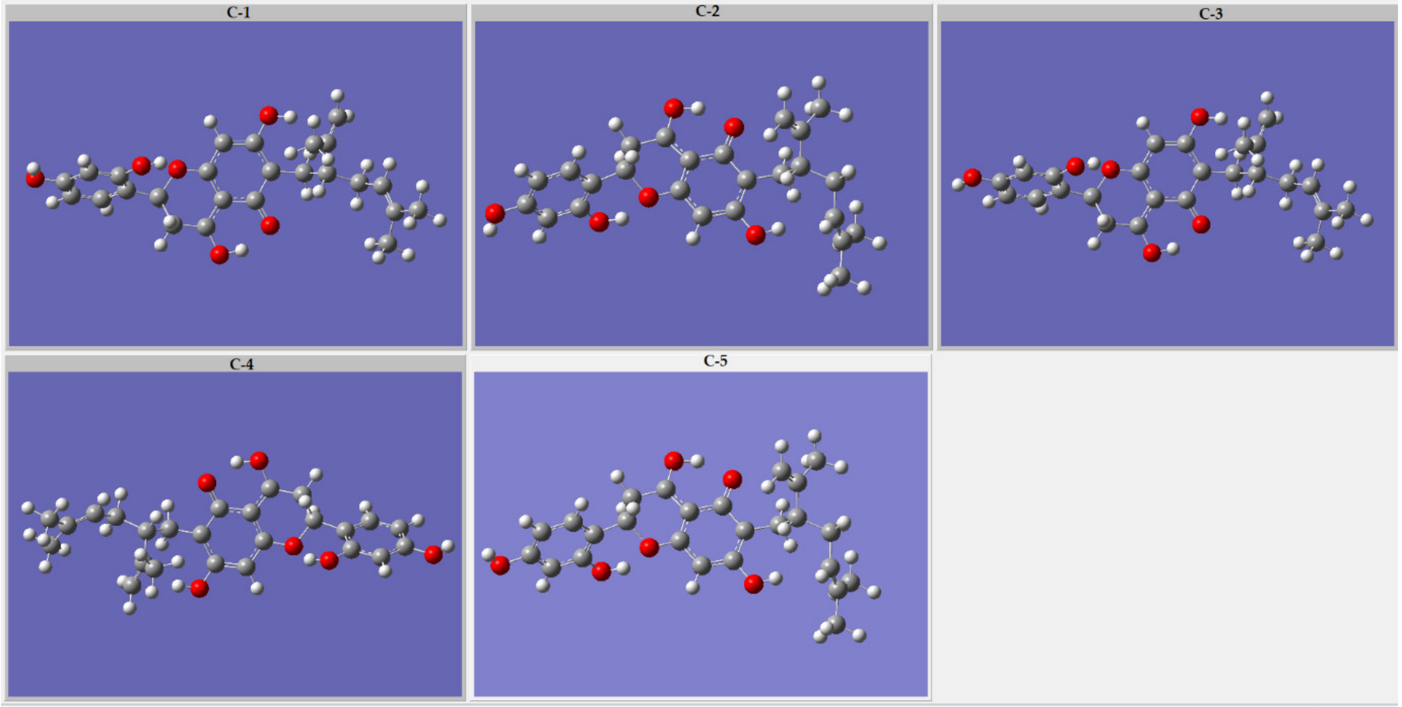

Figure 6S. Optimized conformers of (2*S*,2″*R*)-**2**

Table 4S. Conformational population (%) for the most stable conformers of (2*S*,2″*S*)-**2**

Conformer	Population (%)
C-1	30.59
C-2	2.06
C-3	1.42
C-4	31.24
C-5	31.05
C-6	2.14
C-7	1.50

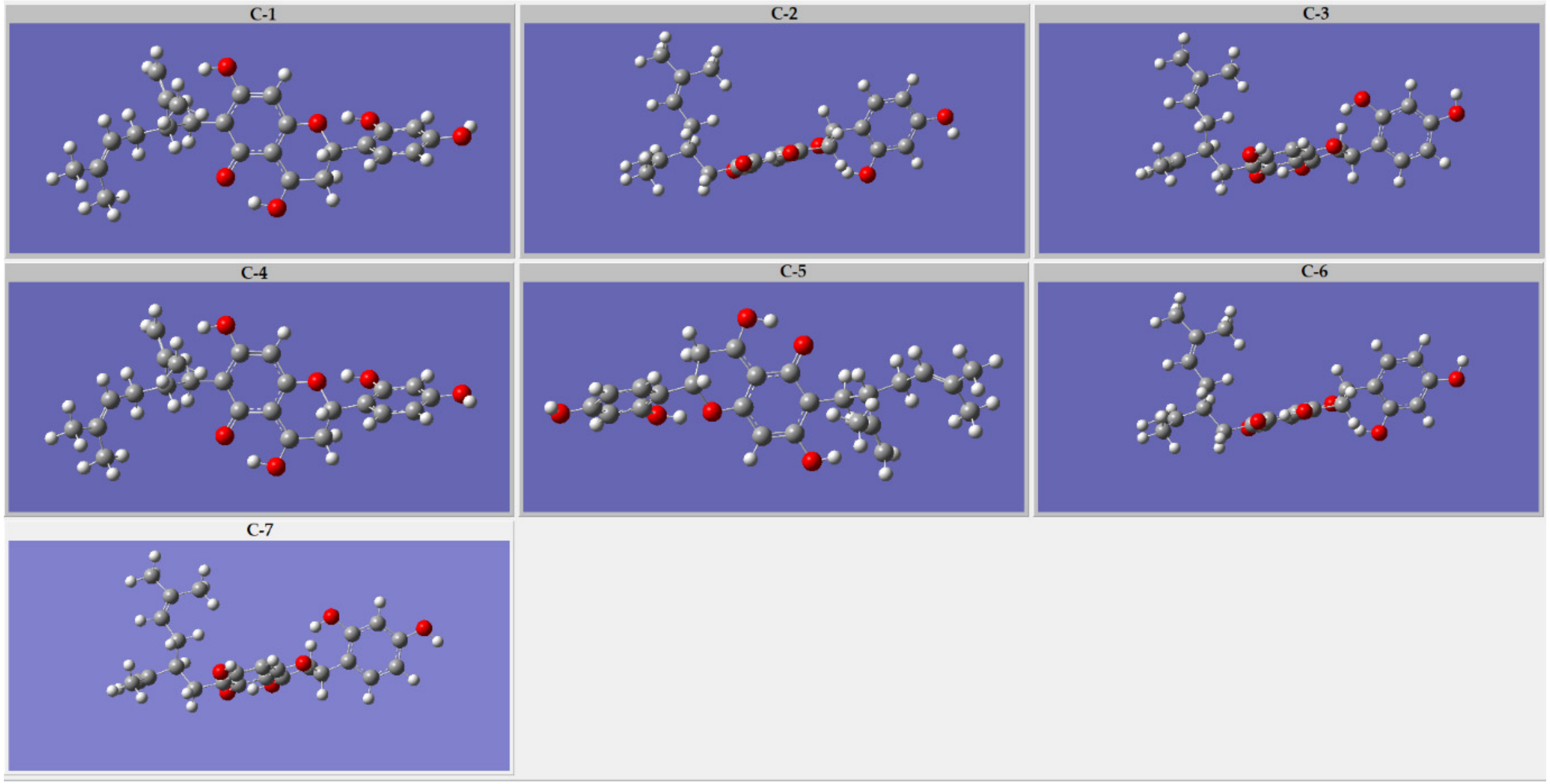

Figure 7S. Optimized conformers of (2*S*,2″*S*)-**2**

Table 5S. Conformational population (%) for the most stable conformers of (2″*S*)-**3**

**Conformer**	**Population (%)**
C-1	53.55
C-2	39.06
C-3	1.36
C-4	1.71
C-5	3.06
C-6	1.26

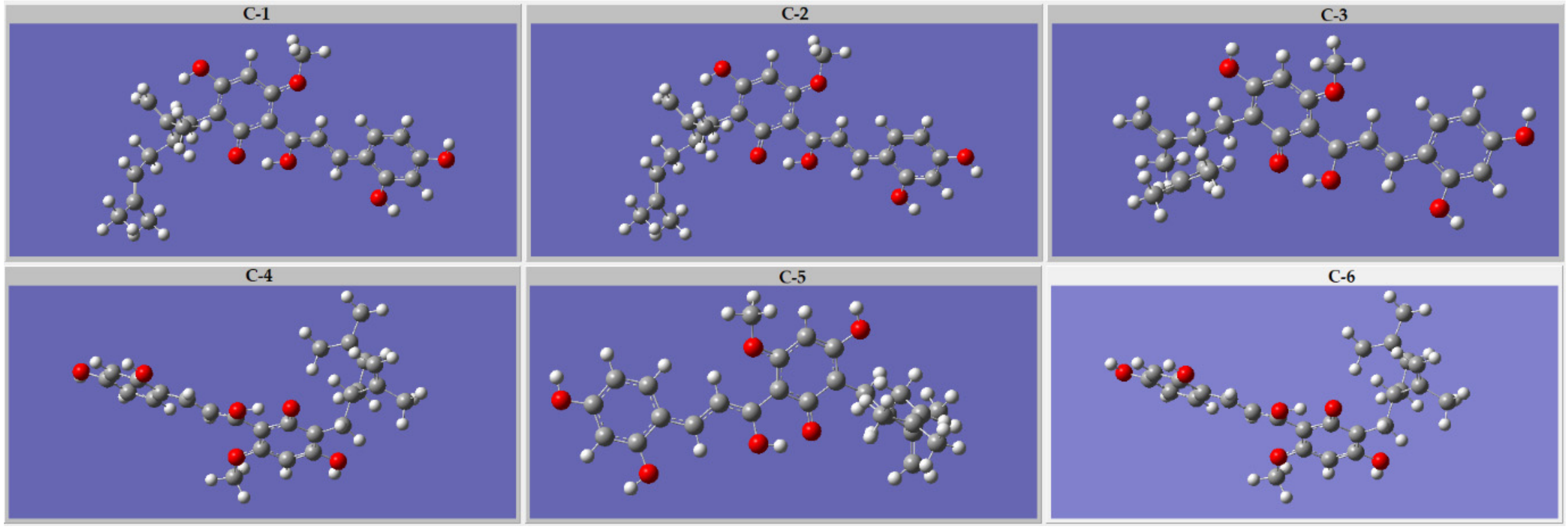

Figure 8S. Optimized conformers of (2″*S*)-**3**

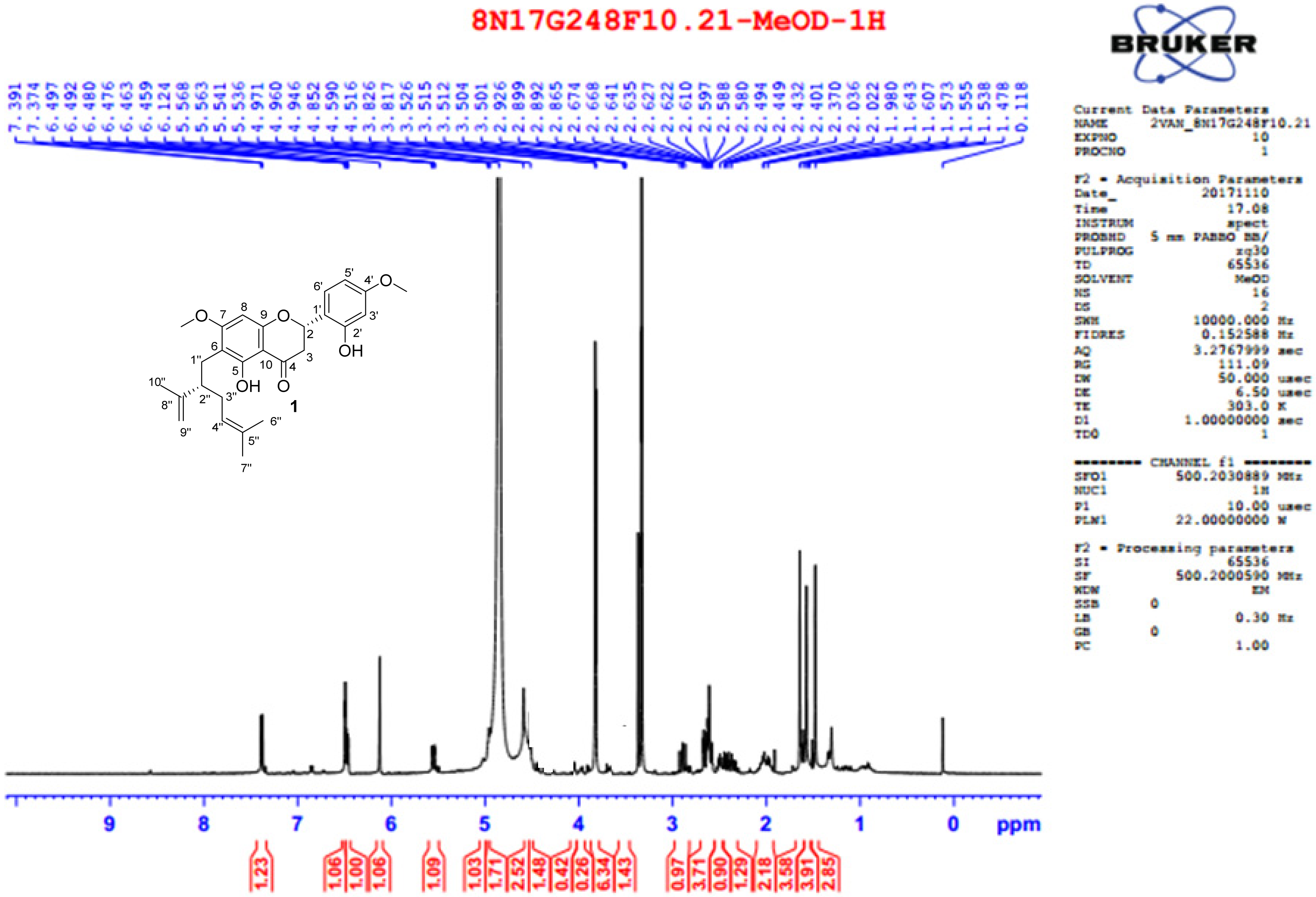

Figure 9S. ^{1}H NMR spectrum of **1** (500 MHz, CD_3OD)

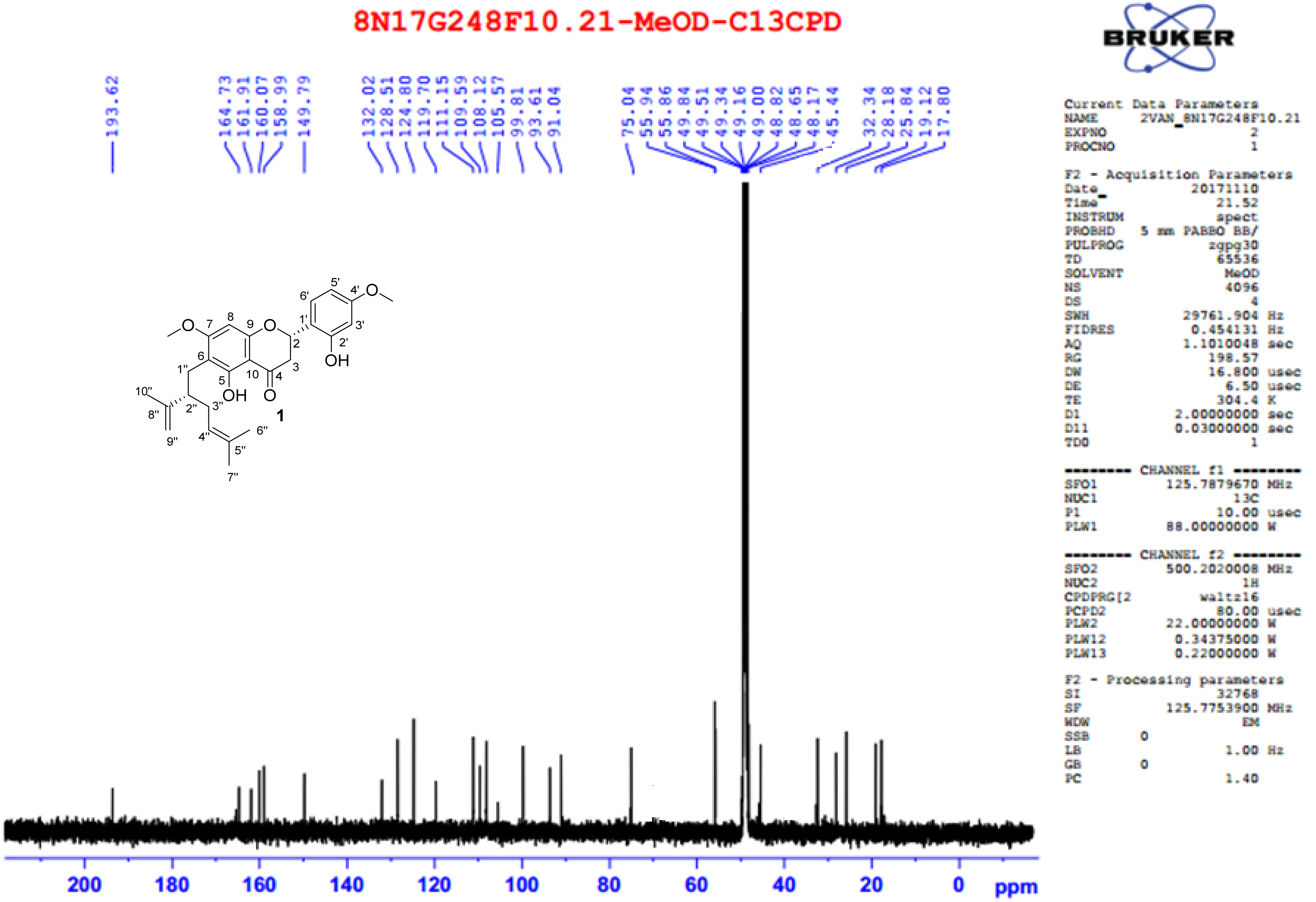

Figure 10S. ^{13}C NMR spectrum of **1** (125 MHz, CD_3OD)

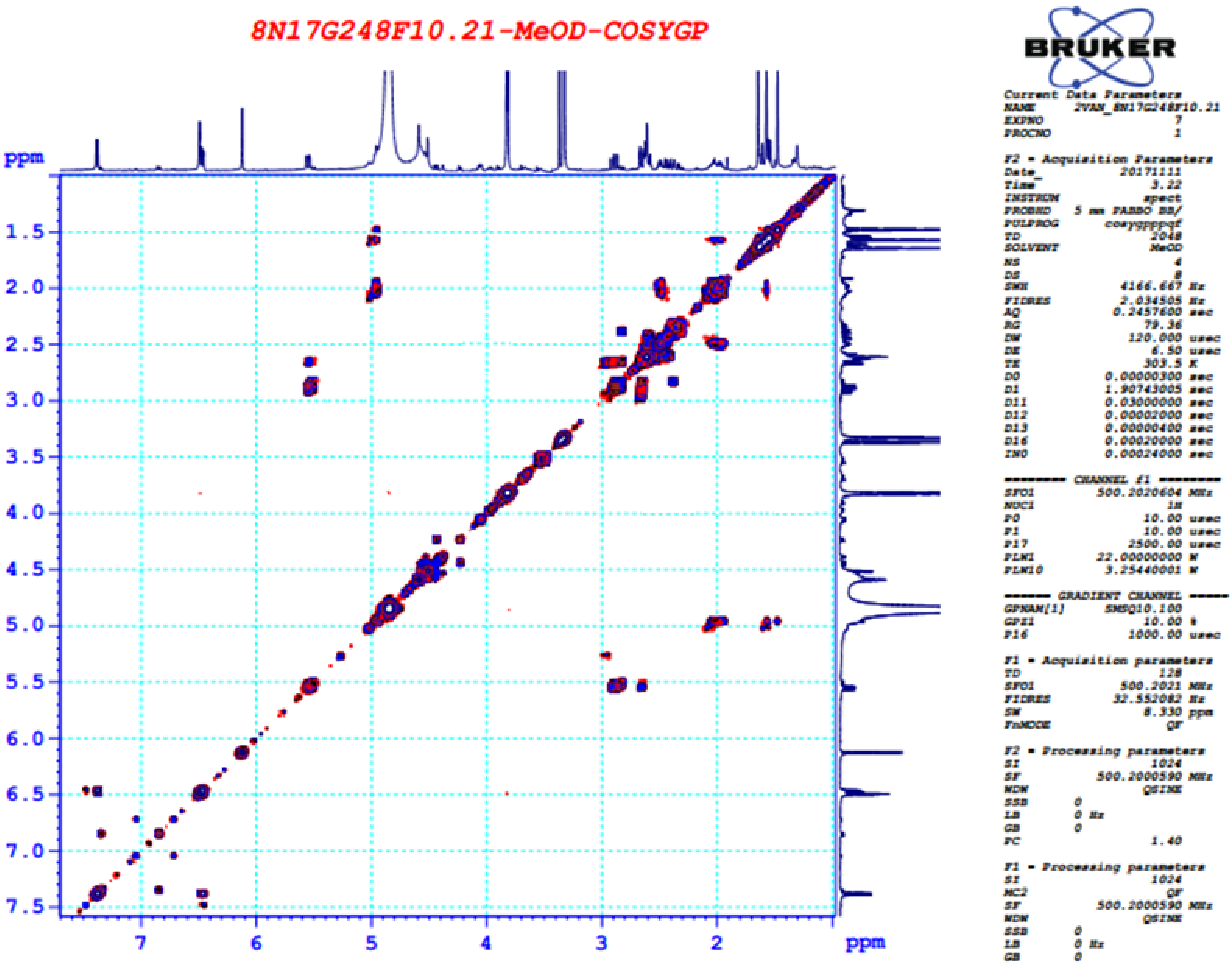

Figure 11S. ^{1}H-^{1}H COSY spectrum of **1** (500 MHz, CD_3OD)

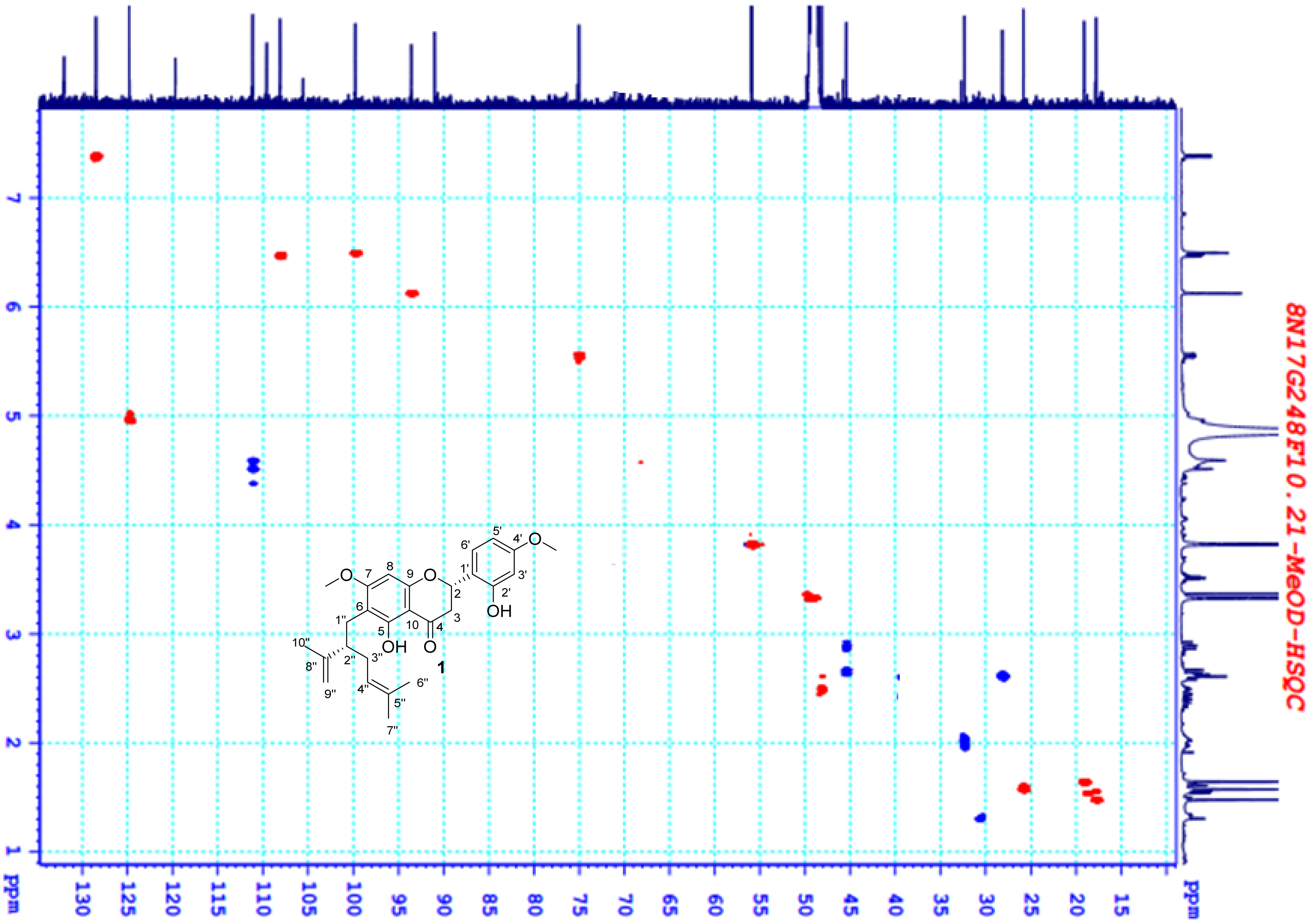

Figure 12S. HSQC spectrum of **1** (^{1}H: 500 MHz, ^{13}C: 125 MHz, CD_3OD)

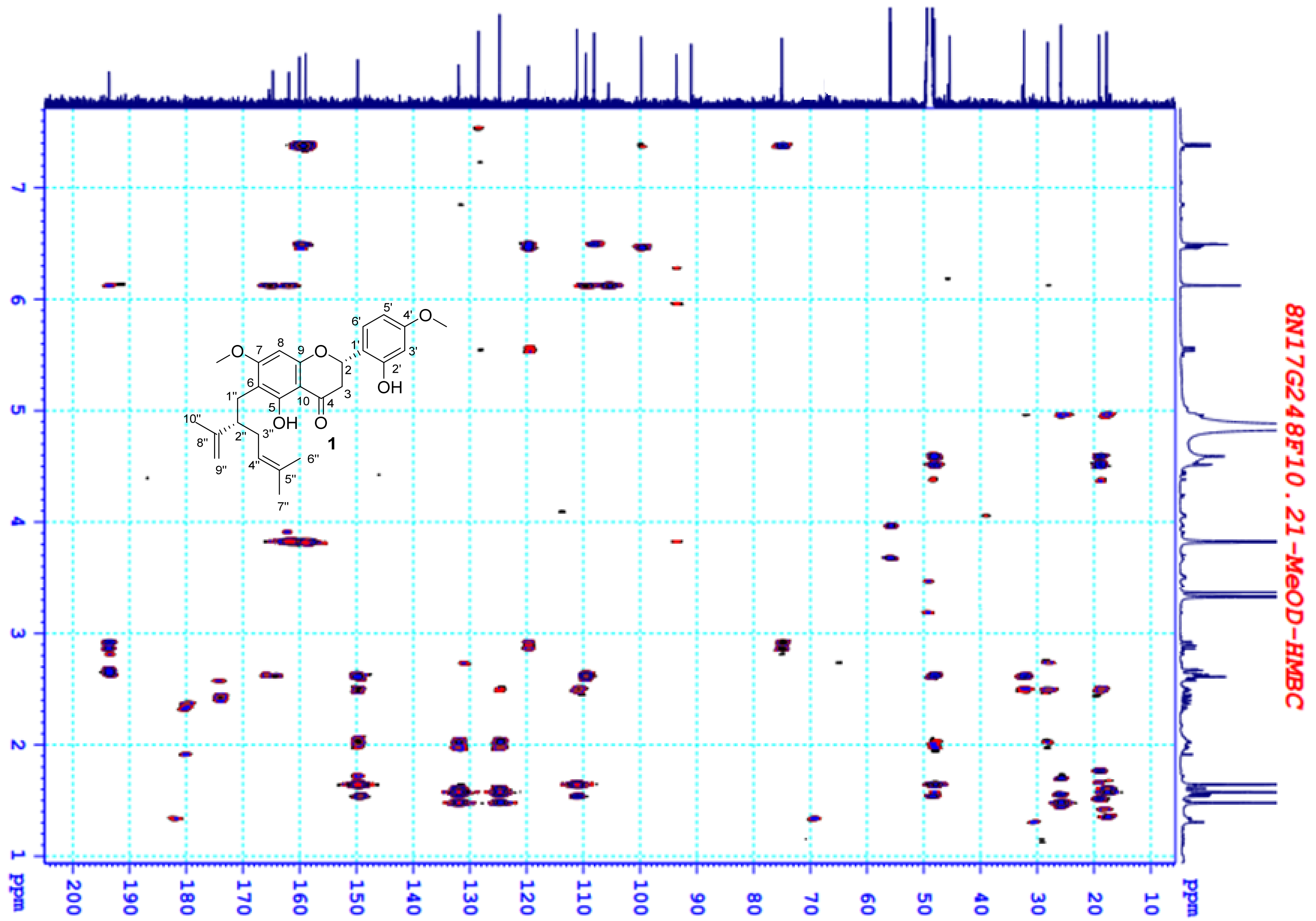

Figure 13S. HMBC spectrum of **1** (^{1}H: 500 MHz, ^{13}C: 125 MHz, CD3OD)

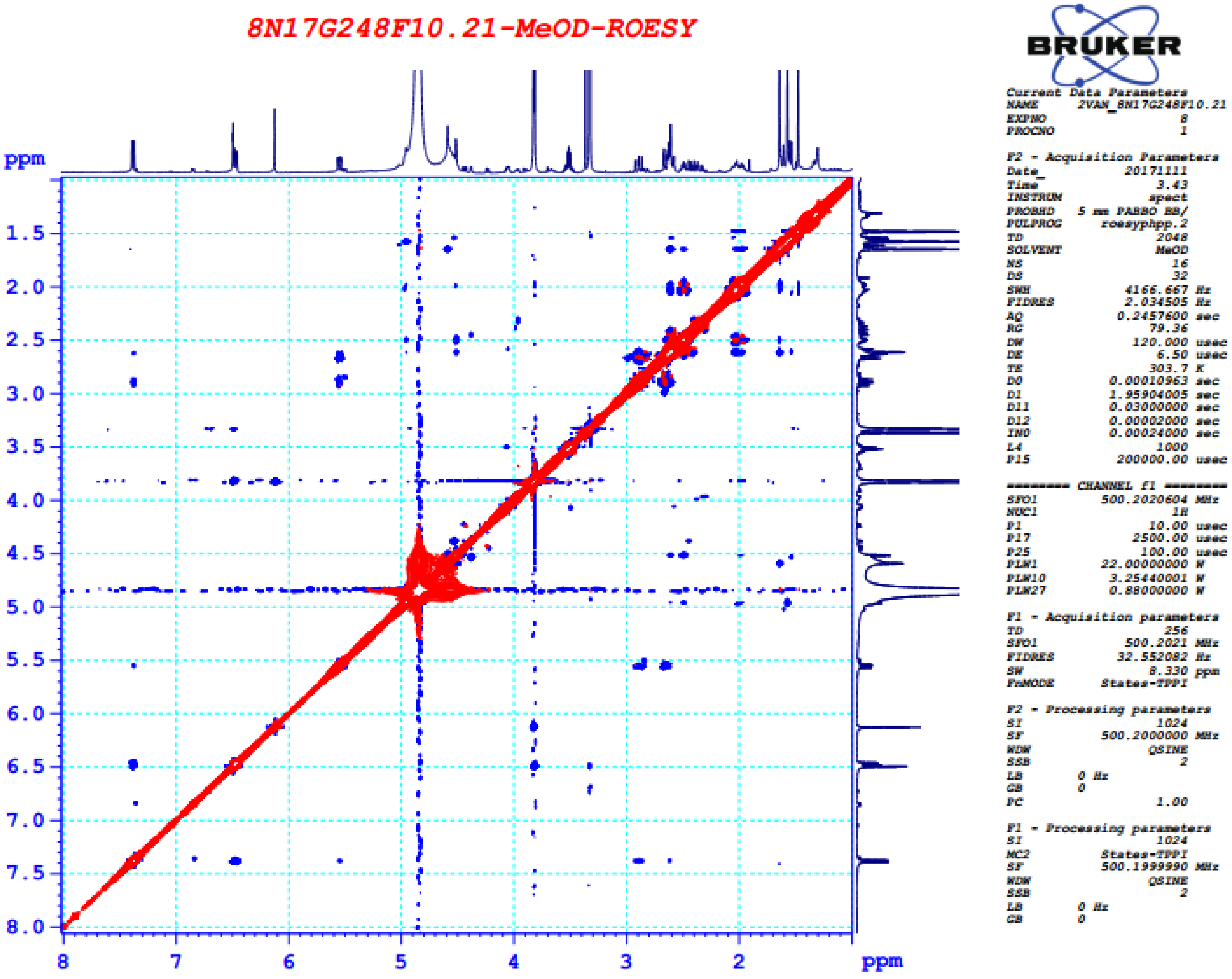

Figure 14S. ROESY spectrum of **1** (500 MHz, CD_3OD)

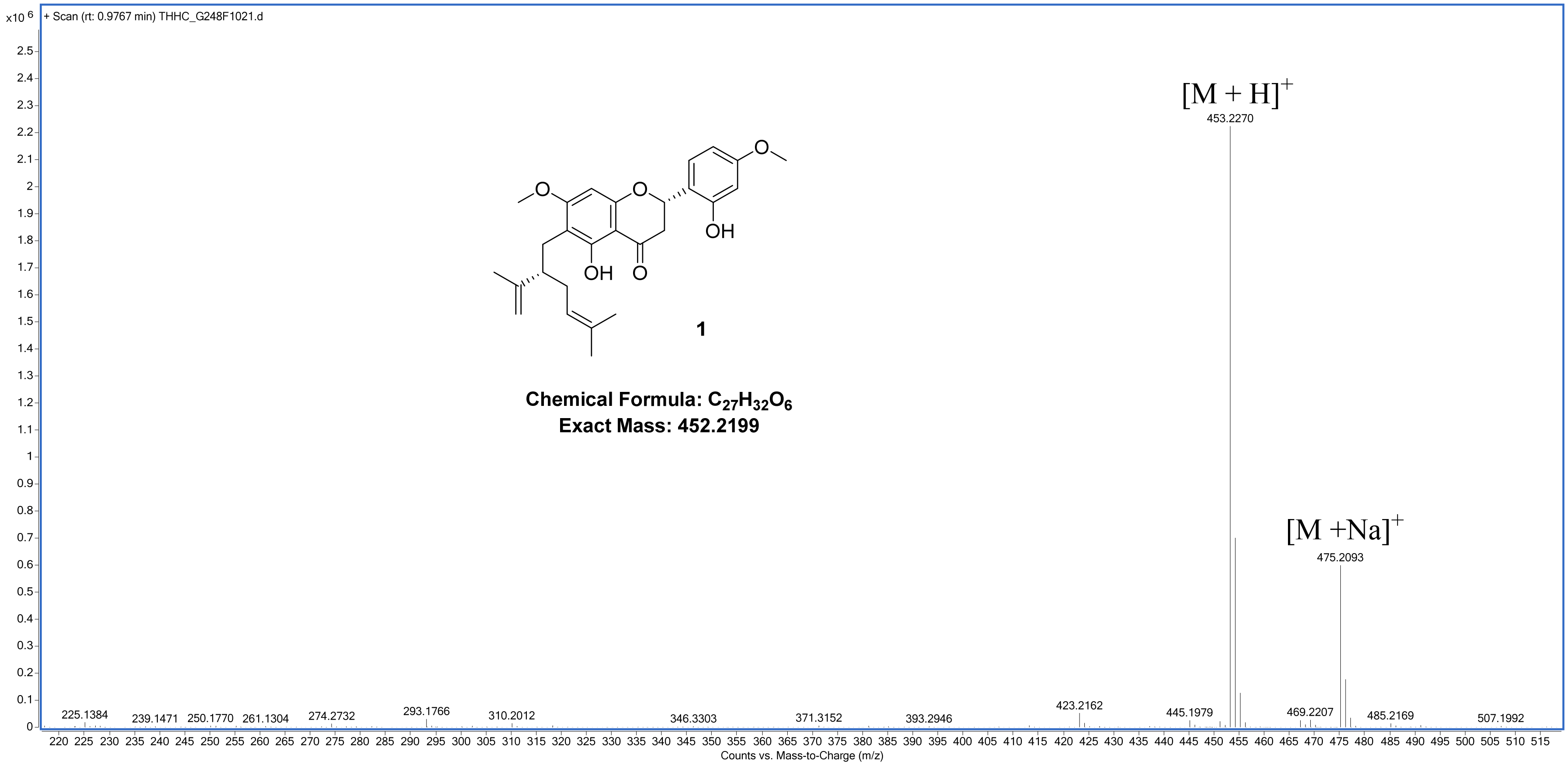

Figure 15S. HR-ESI-MS of **1**

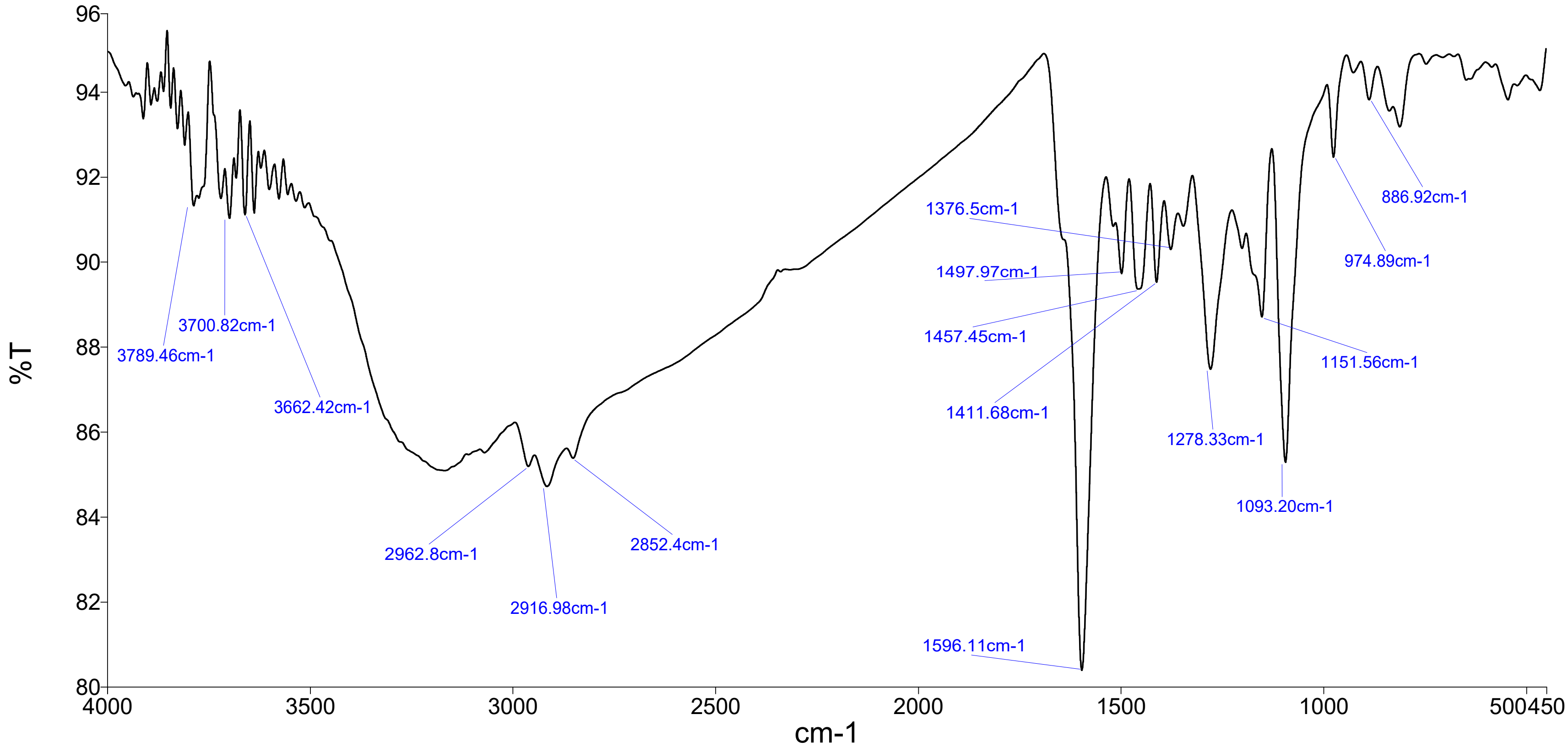

Figure 16S. IR spectrum of **1**

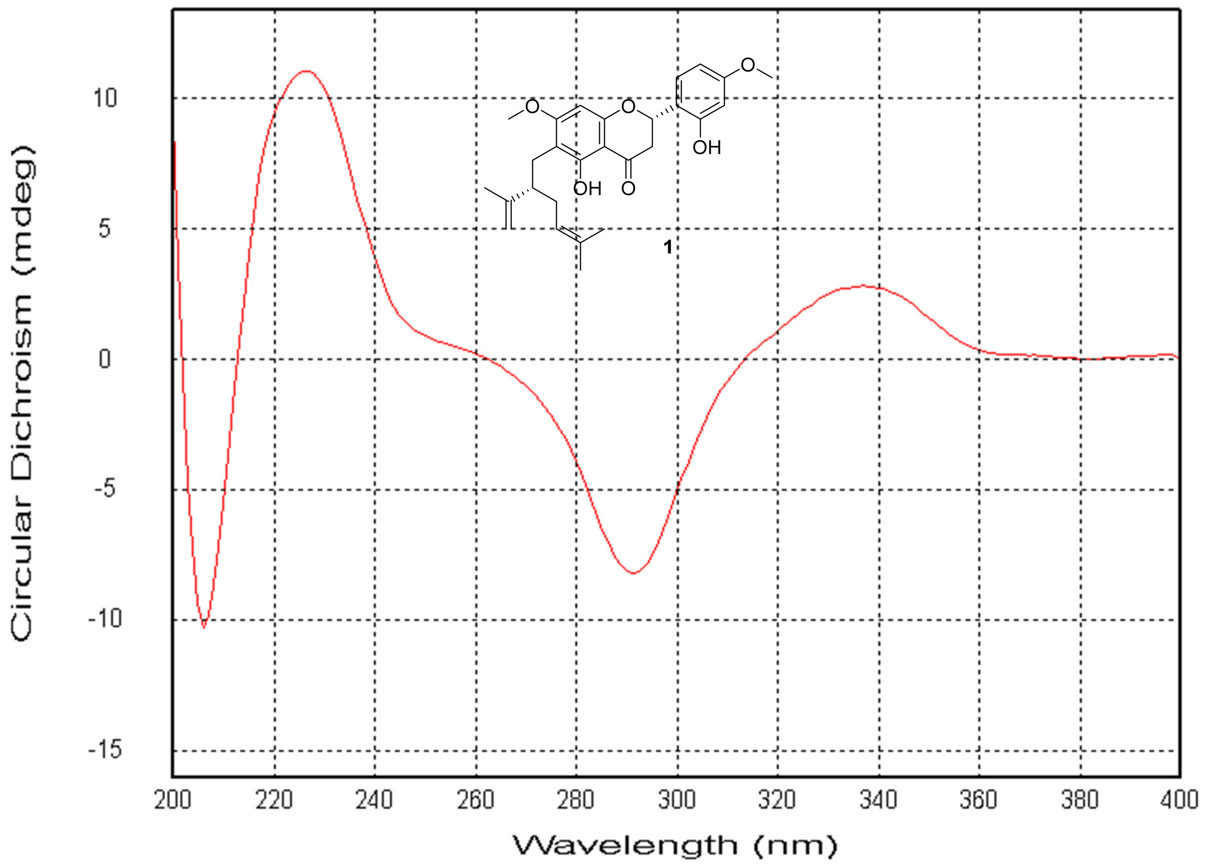

Figure 17S. CD spectrum of **1**

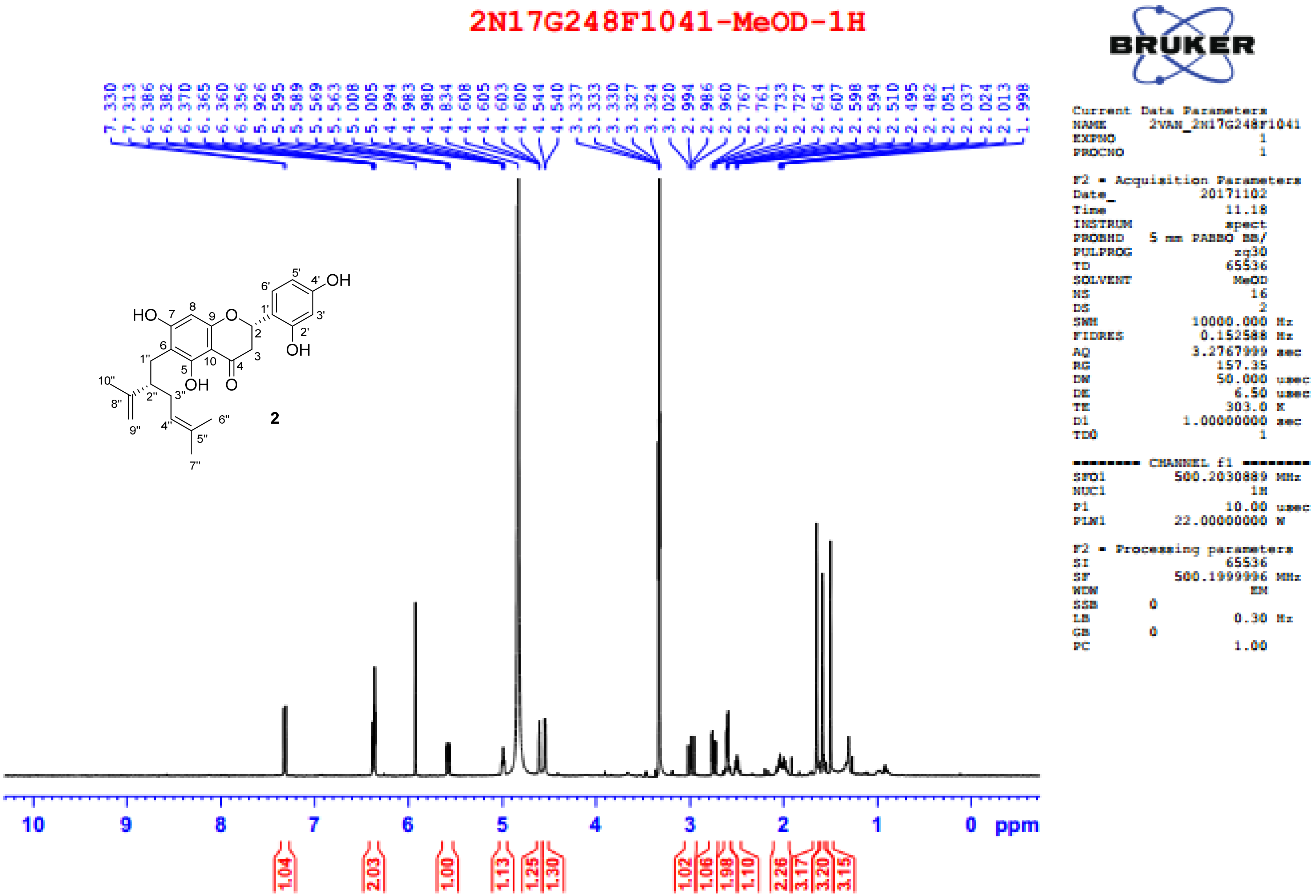

Figure 18S. ^{1}H NMR spectrum of **2** (500 MHz, CD_3OD)

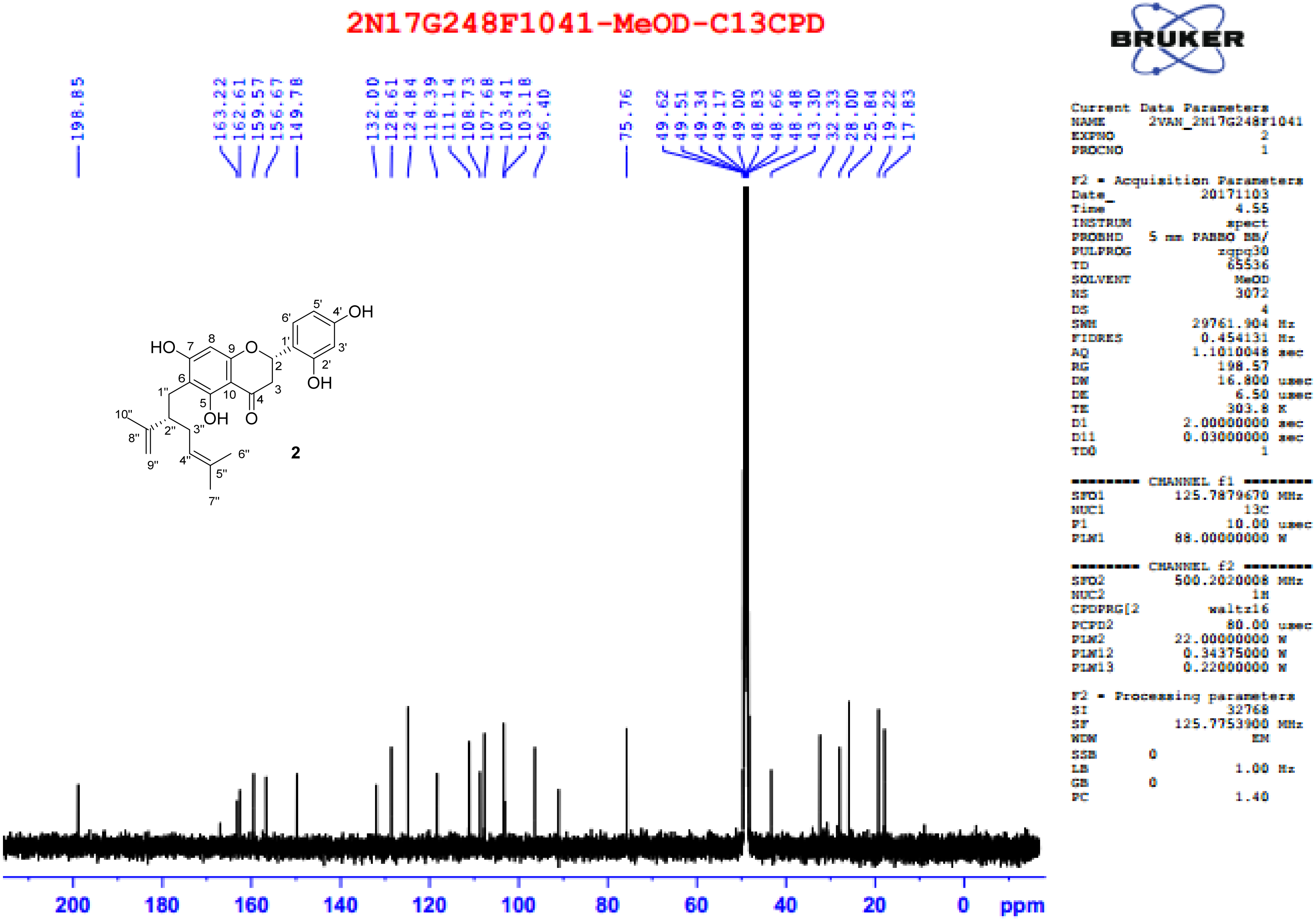

Figure 19S. ^{13}C NMR spectrum of **2** (125 MHz, CD_3OD)

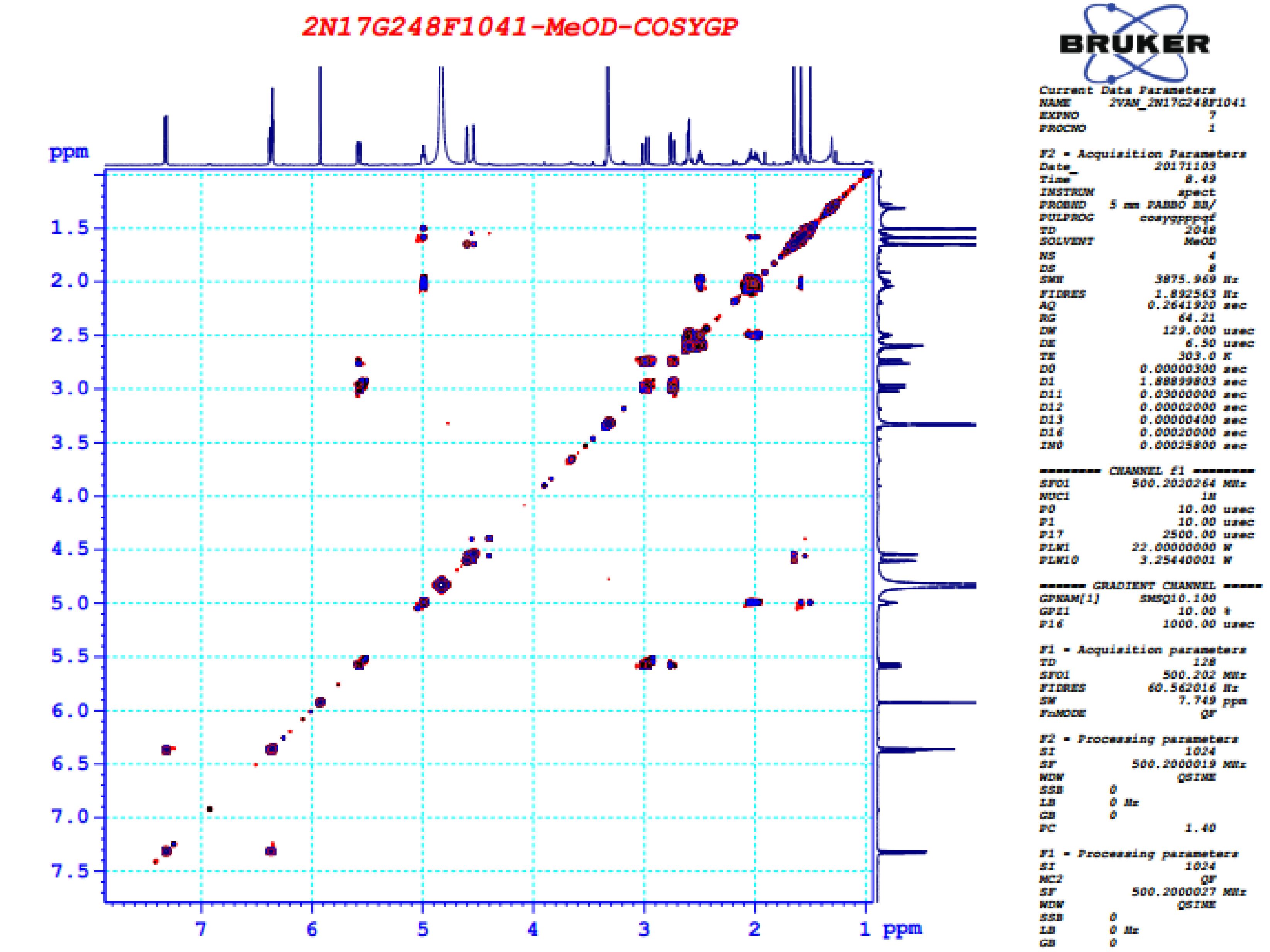

Figure 20S. ^{1}H-^{1}H COSY spectrum of **2** (500 MHz, CD_3OD)

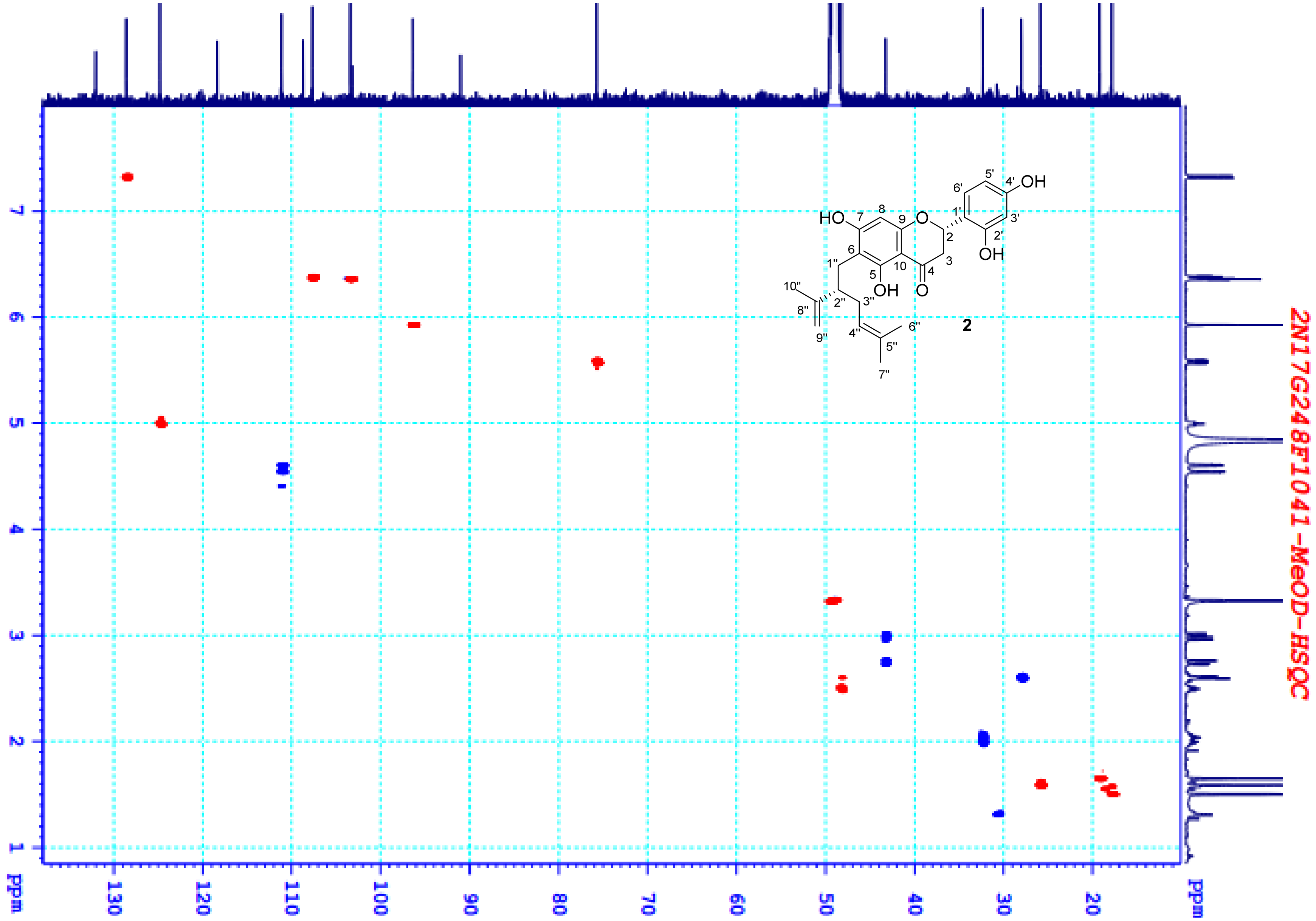

Figure 21S. HSQC spectrum of **2** (^{1}H: 500 MHz, ^{13}C: 125 MHz, CD_3OD)

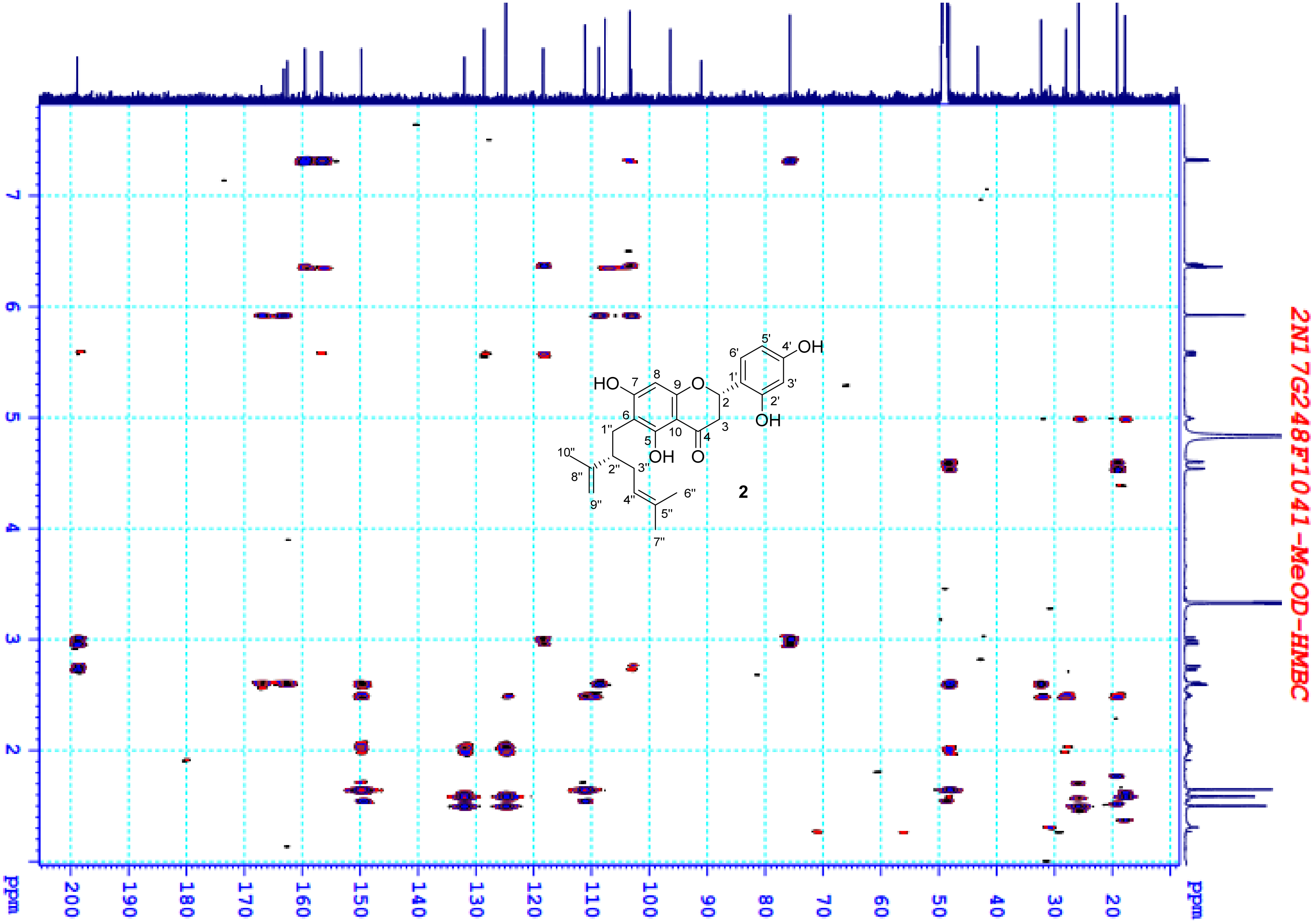

Figure 22S. HMBC spectrum of **2** (^{1}H: 500 MHz, ^{13}C: 125 MHz, CD_3OD)

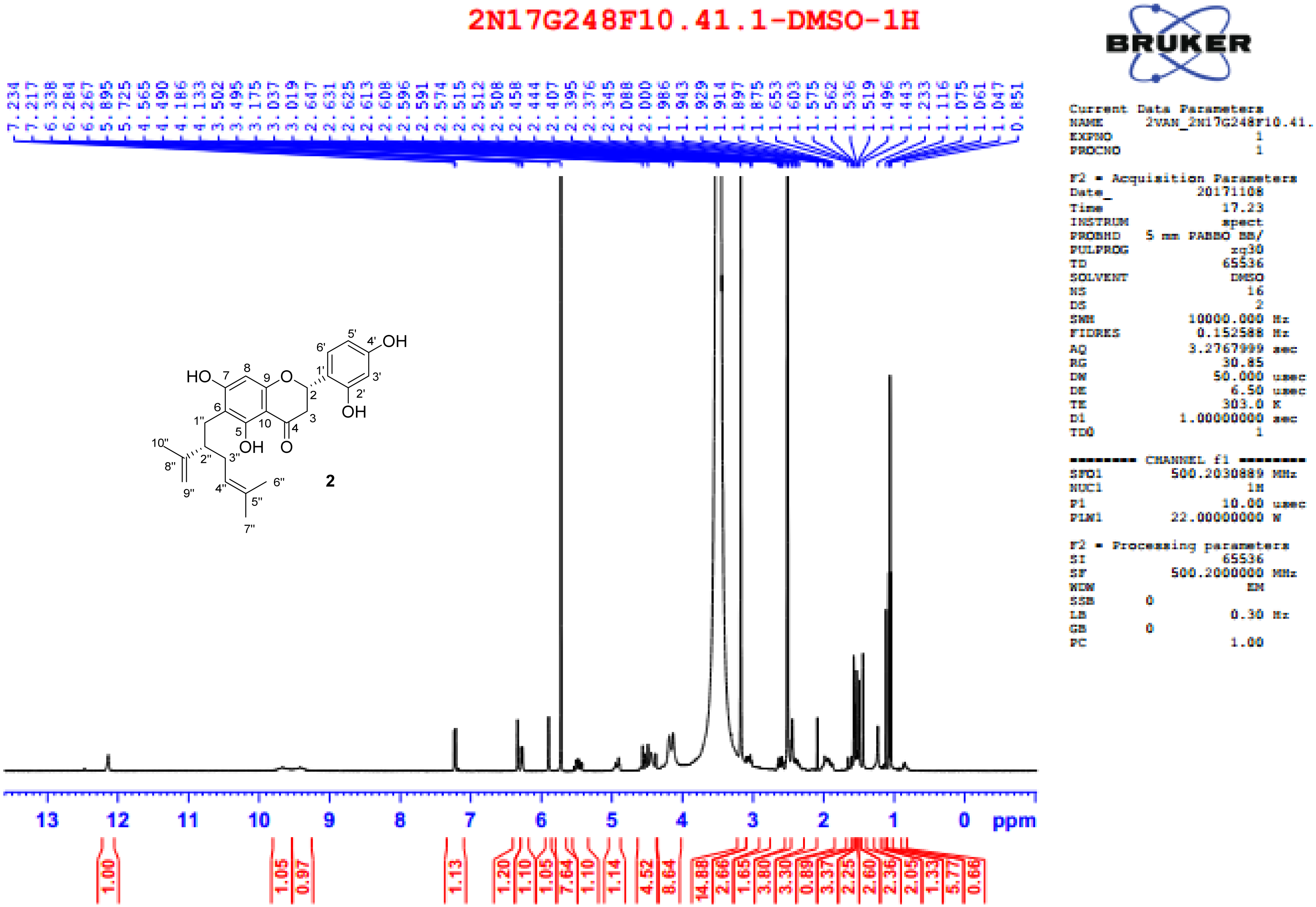

Figure 23S. ^{1}H NMR spectrum of **2** (500 MHz, DMSO-d_6)

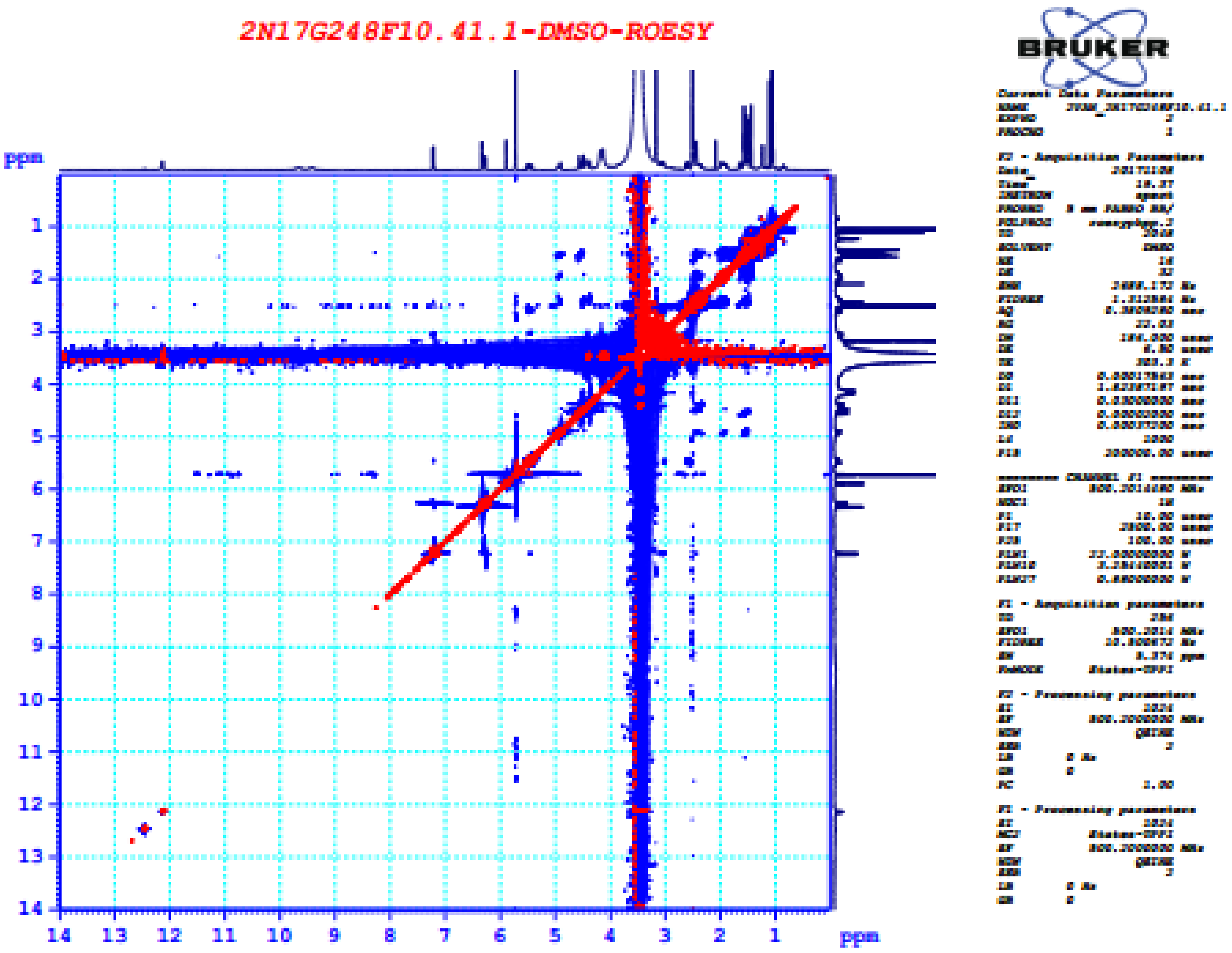

Figure 24S. ROESY spectrum of **2** (500 MHz, DMSO-d_6)

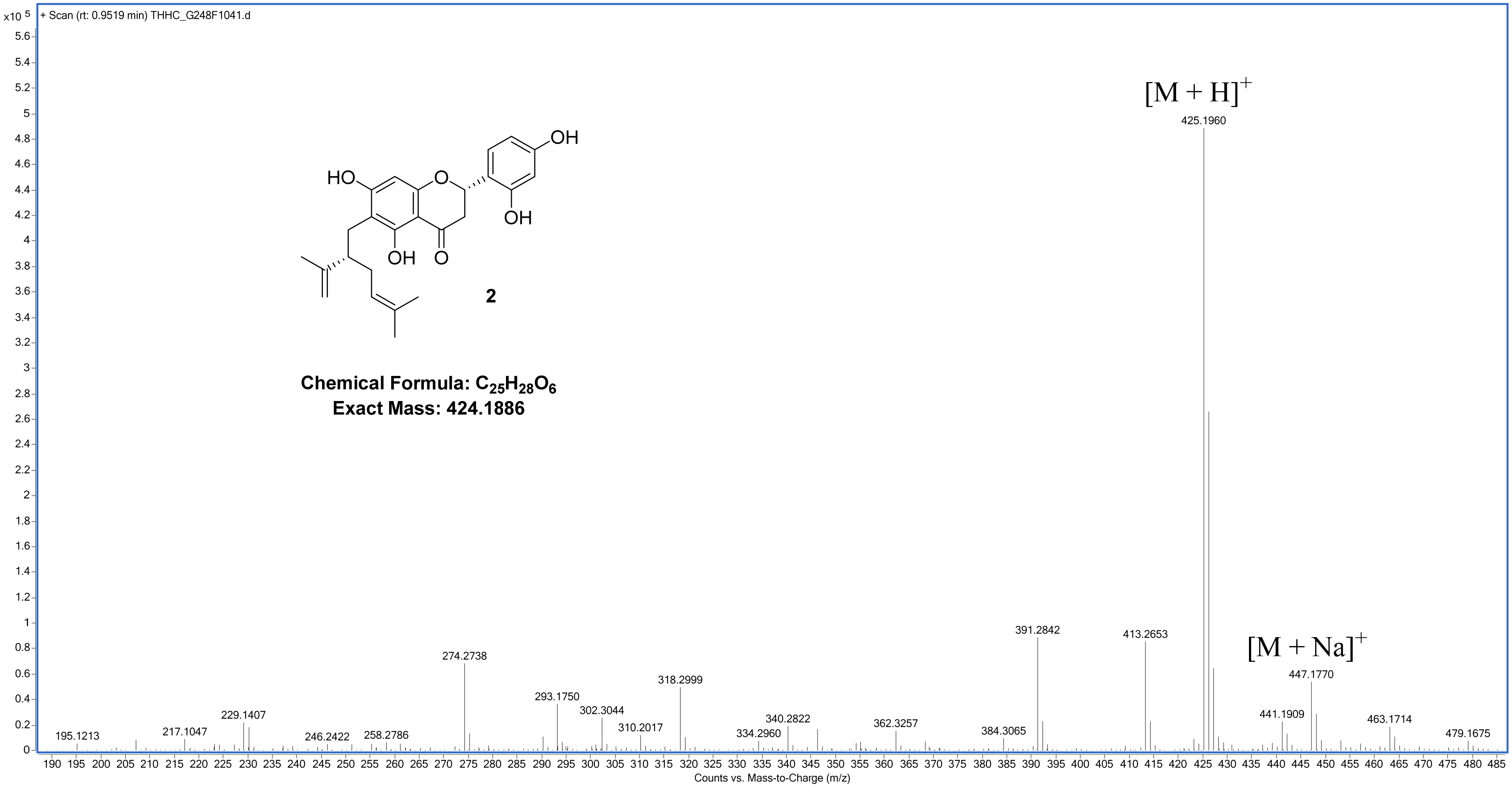

Figure 25S. HR-ESI-MS of **2**

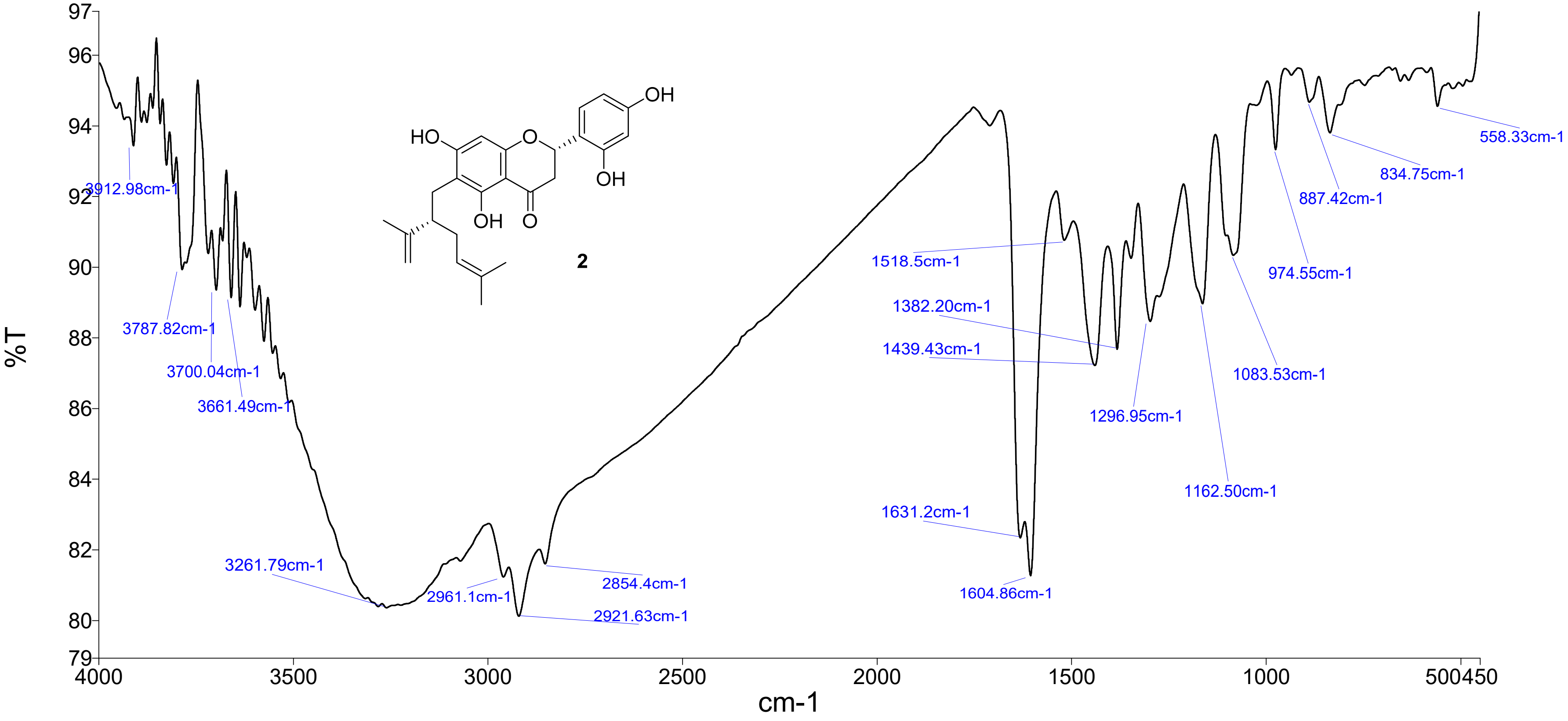

Figure 26S. IR spectrum of **2**

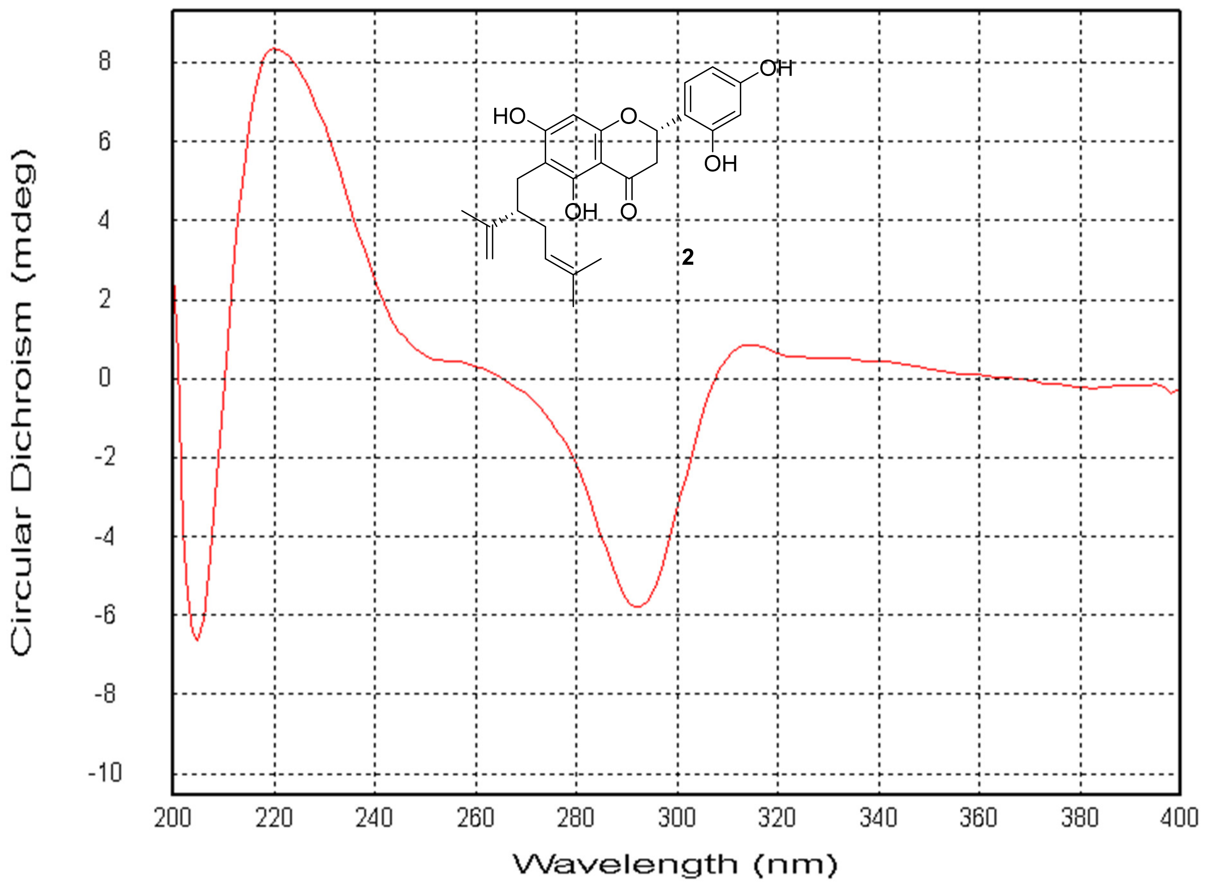

Figure 27S. CD spectrum of **2**

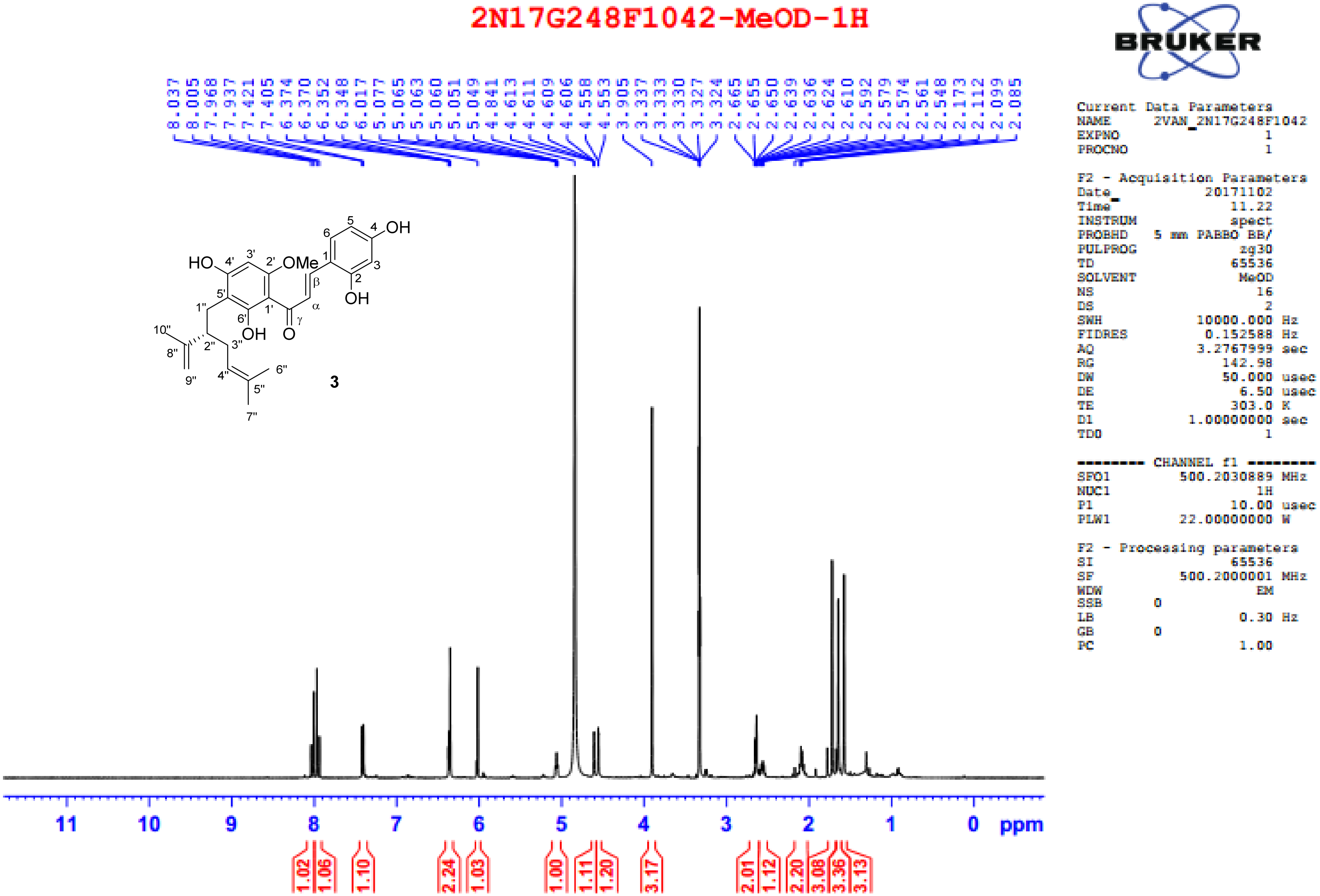

Figure 28S. ^{1}H NMR spectrum of **3** (500 MHz, CD_3OD)

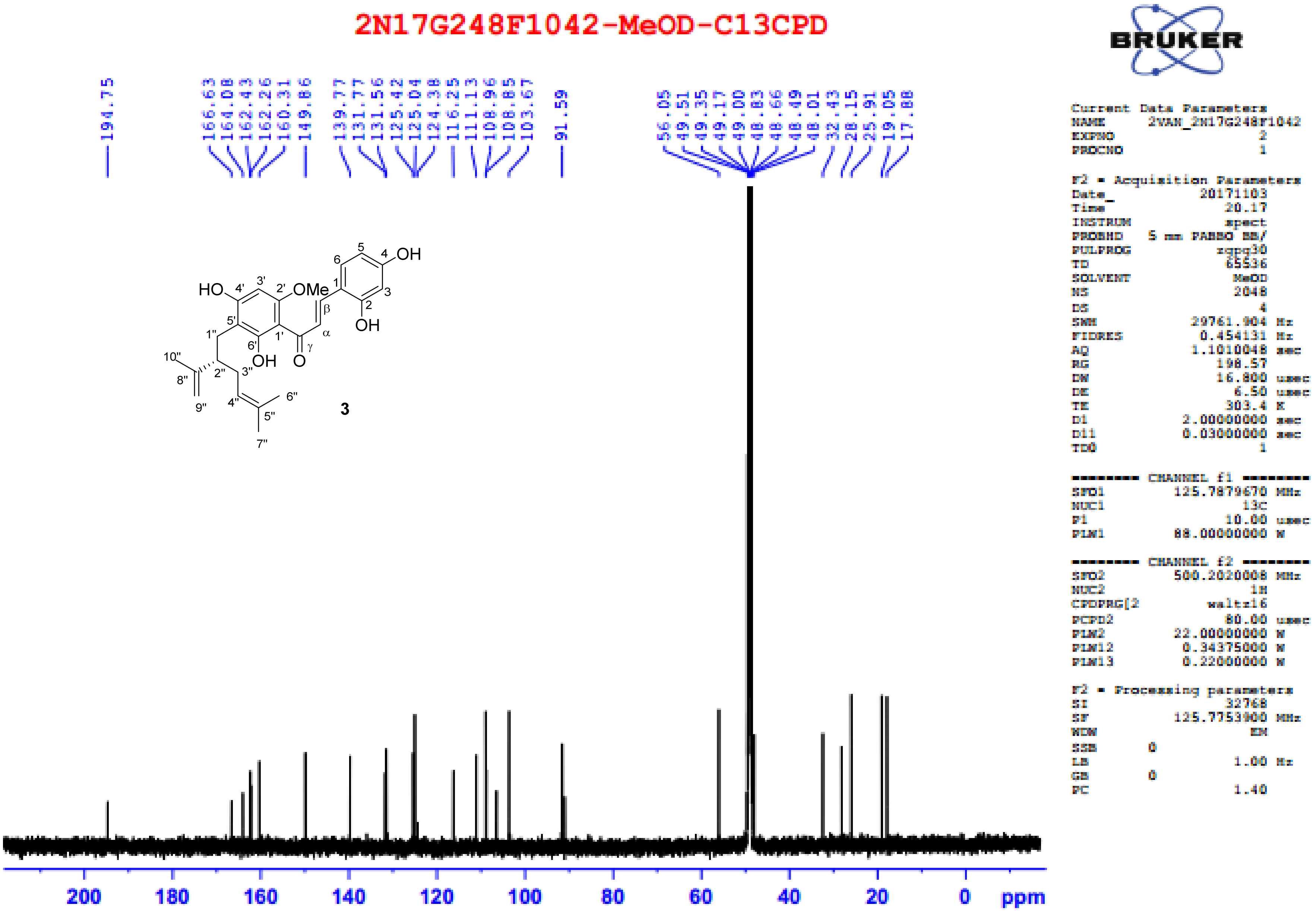

Figure 29S. ^{13}C NMR spectrum of **3** (125 MHz, CD_3OD)

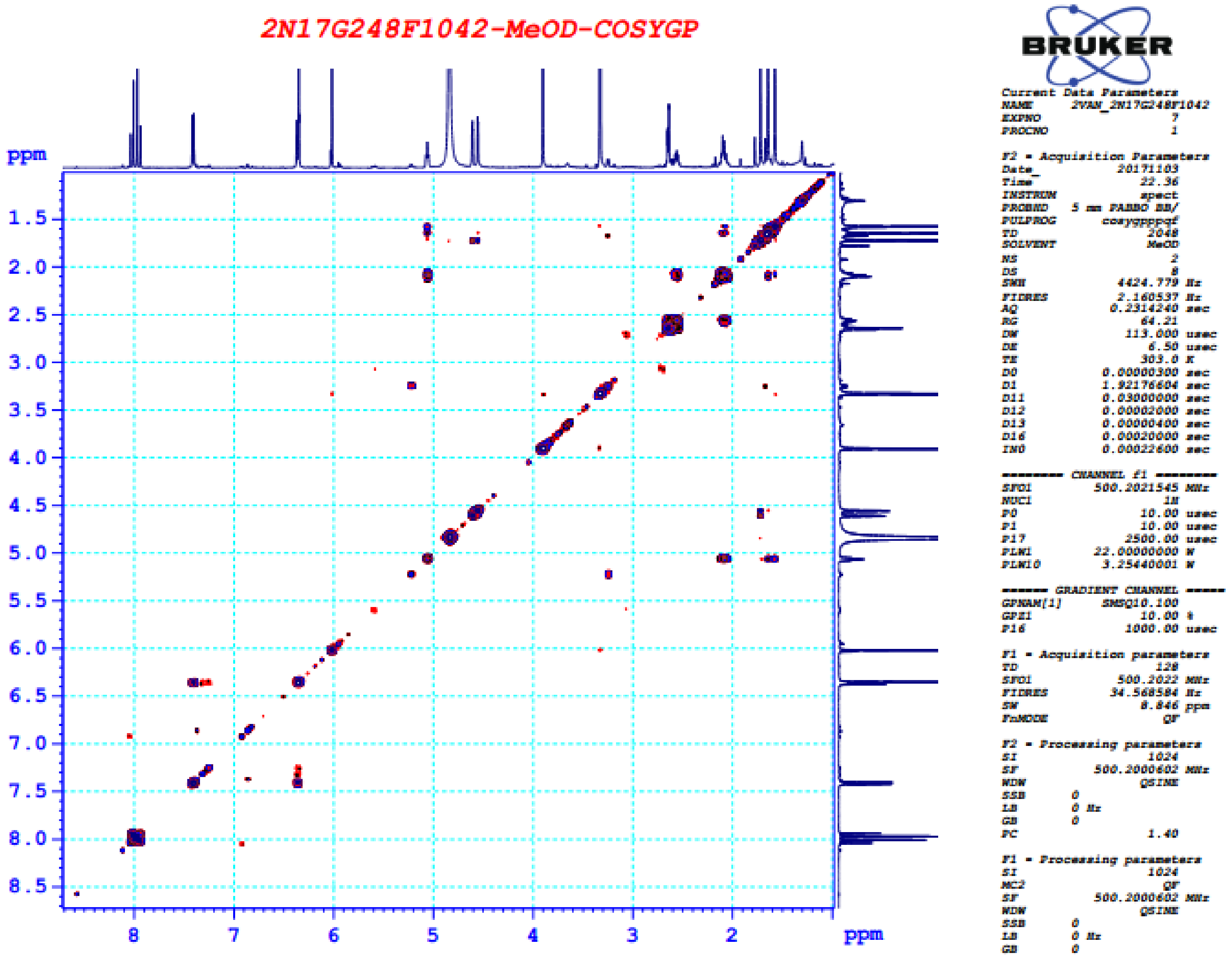

Figure 30S. ^{1}H-^{1}H COSY spectrum of **3** (500 MHz, CD_3OD)

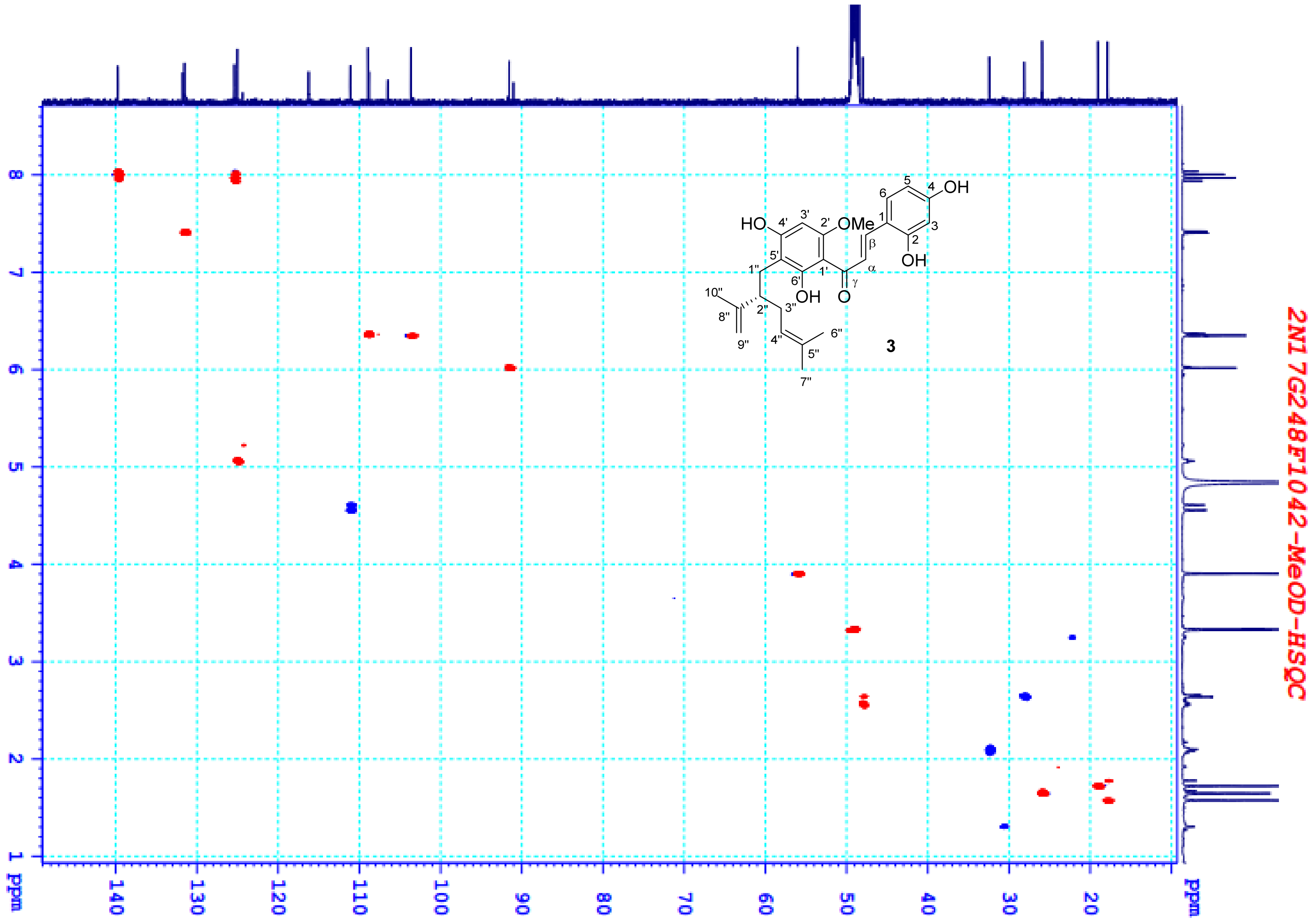

Figure 31S. HSQC spectrum of **3** (^{1}H: 500 MHz, ^{13}C: 125 MHz, CD_3OD)

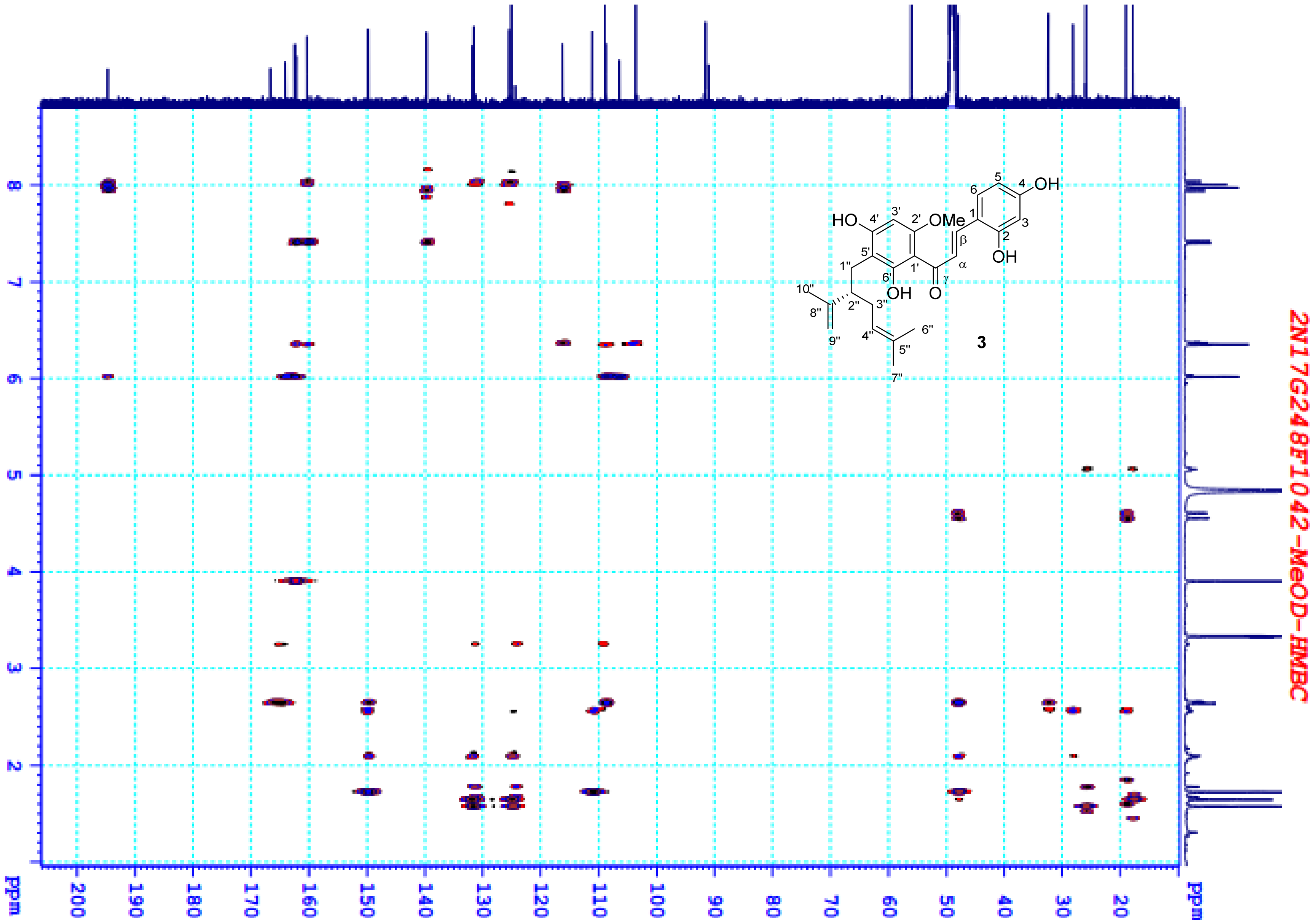

Figure 32S. HMBC spectrum of **3** (^{1}H: 500 MHz, ^{13}C: 125 MHz, CD_3OD)

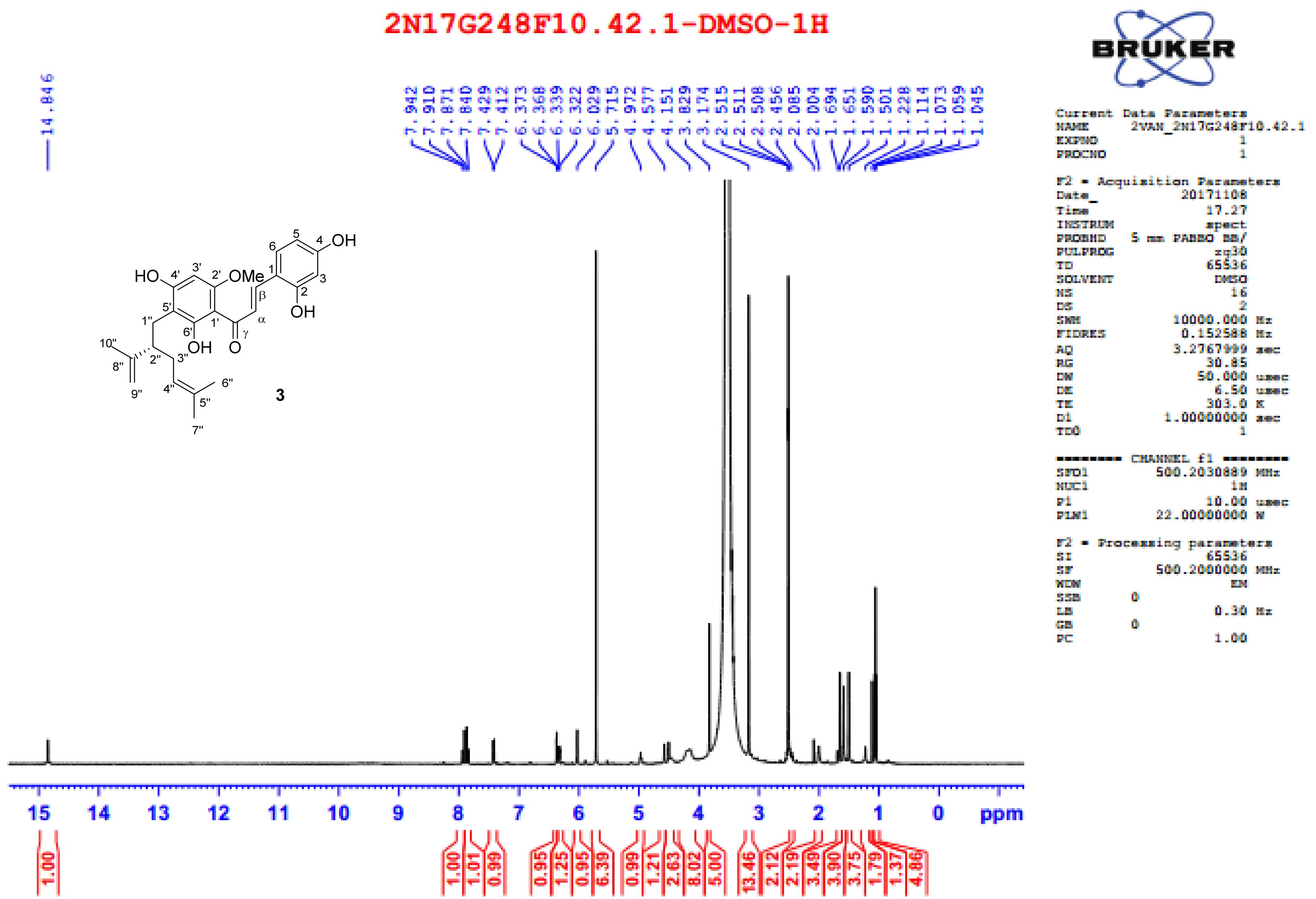

Figure 33S. ^{1}H-NMR spectrum of **3** (500 MHz, DMSO-d_6)

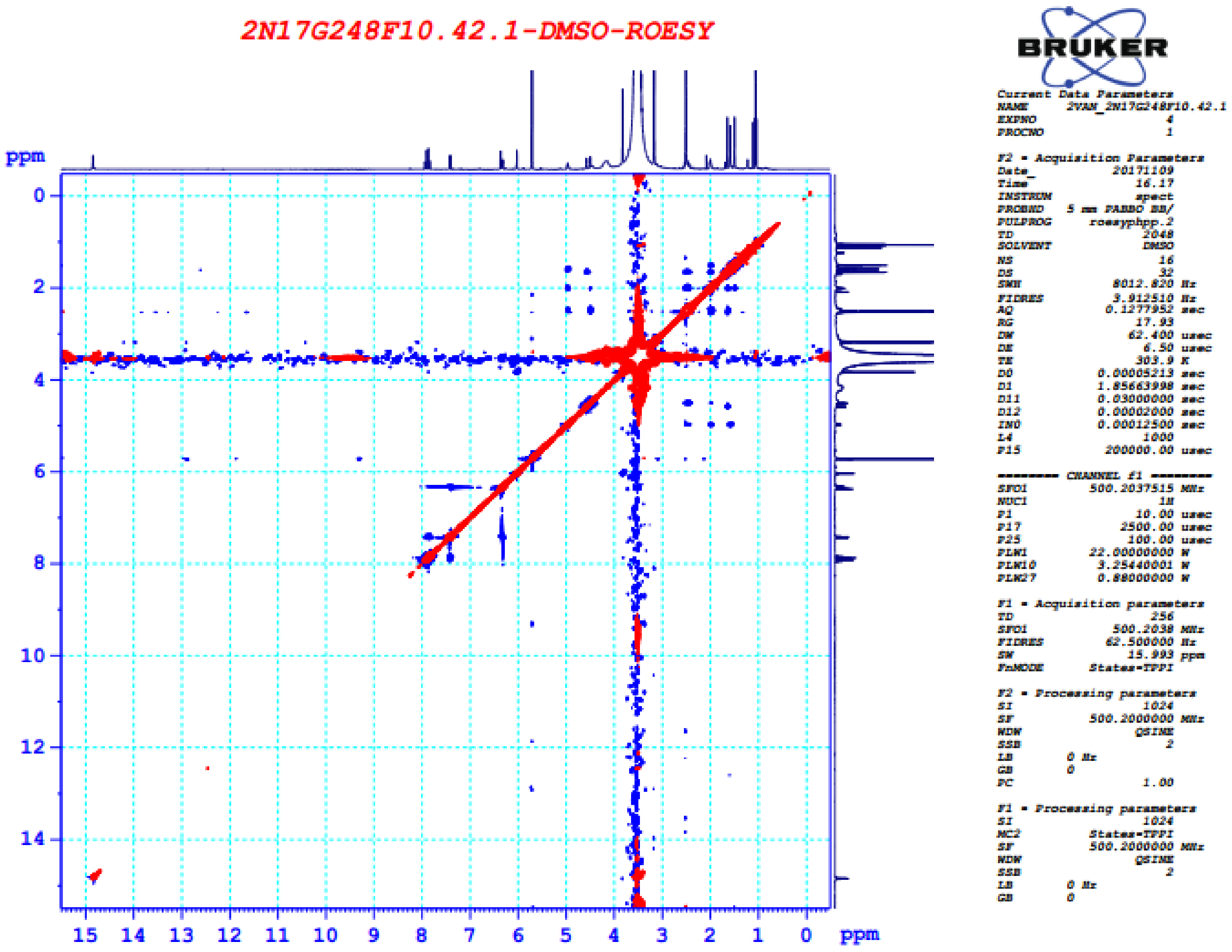

Figure 34S. ROESY spectrum of **3** (500 MHz, DMSO-d_6)

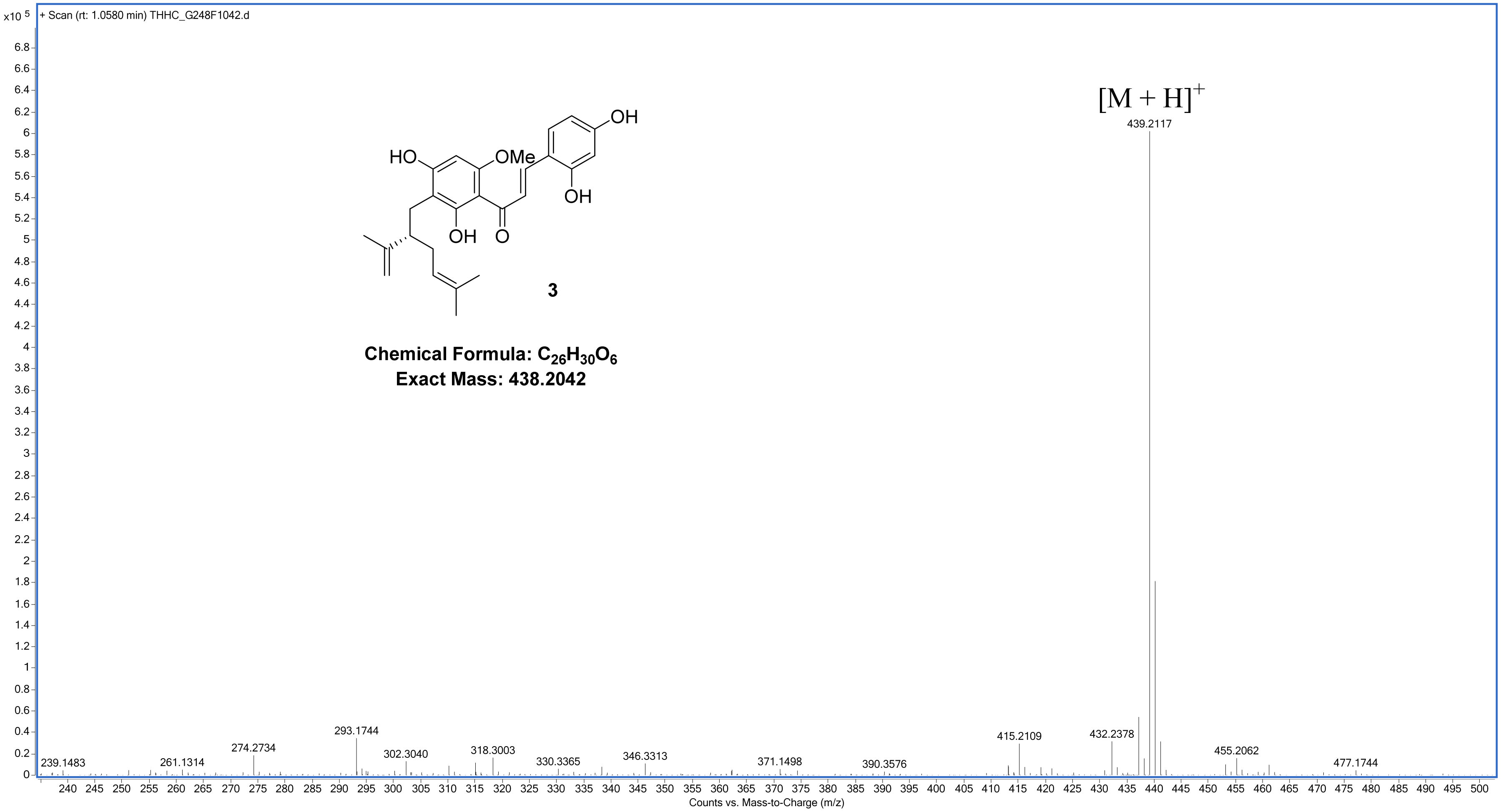

Figure 35S. HR-ESI-MS of **3**

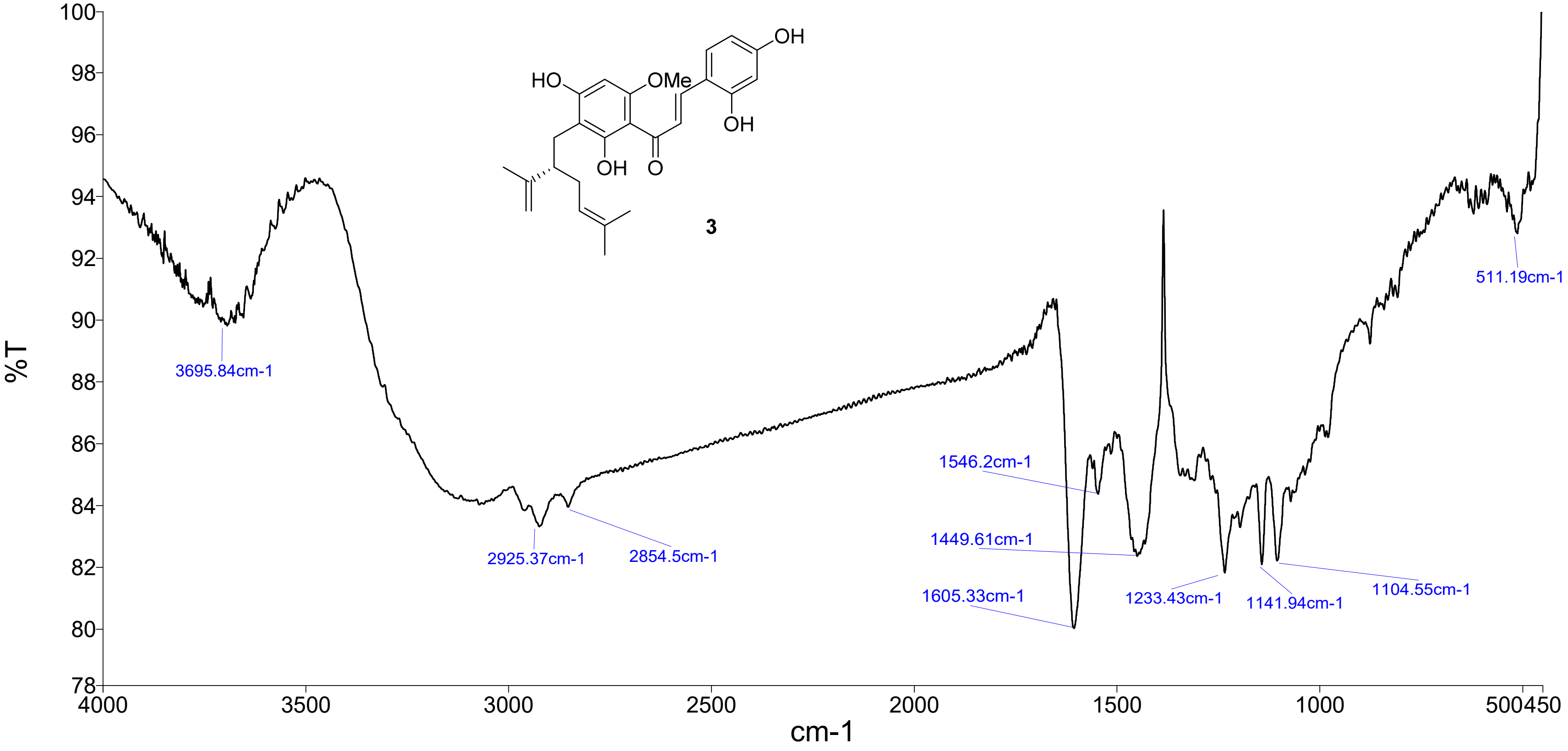

Figure 36S. IR spectrum of **3**

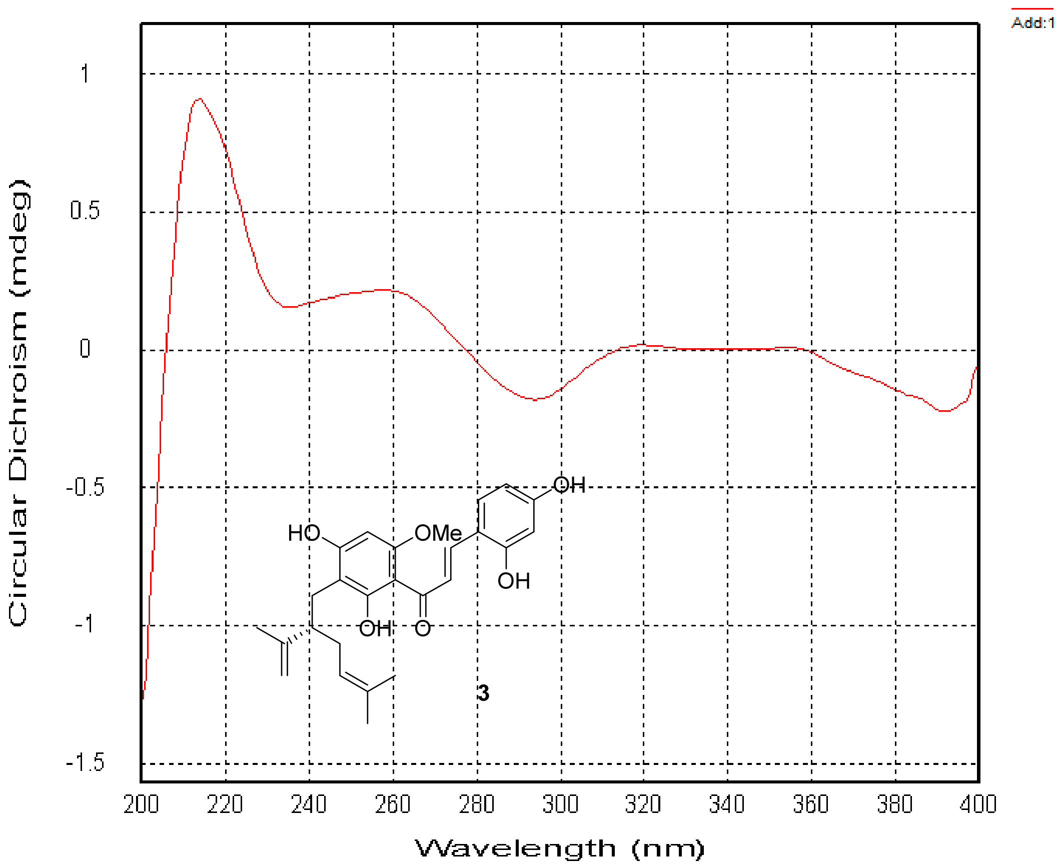

Figure 37S. CD spectrum of **3**
